# Real-world analysis of survival benefit of surgery and adjuvant therapy in elderly patients with colorectal cancer

**DOI:** 10.1038/s41598-023-41713-1

**Published:** 2023-09-08

**Authors:** Lei Zhang, Qixin Li, Chenhao Hu, Zhe Zhang, Junjun She, Feiyu Shi

**Affiliations:** 1 Department of General Surgery, The First Affiliated Hospital of Xi’an Jiaotong University, 277 Yanta West Road, Xi’an, Shaanxi China; 2 Center for Gut Microbiome Research, Med-X Institute, The First Affiliated Hospital of Xi’an Jiaotong University, Xi’an, Shaanxi China

**Keywords:** Cancer, Cancer therapy, Gastrointestinal cancer

## Abstract

Treatment guidelines for colorectal cancer (CRC) in elderly patients remain unclear. This study aimed to investigate whether elderly patients (≥ 70 years) with CRC benefit from surgery and adjuvant therapy. A total of 90,347 eligible CRC patients older than 70 years were collected from the Surveillance, Epidemiology, and End Results (SEER) database and divided into a surgery group and a no-surgery group. After being matched by propensity score matching at a 1:1 ratio, 23,930 patients were included in our analysis. The Kaplan‒Meier method and log-rank test were applied to compare overall survival (OS) and cancer-specific survival (CSS). Univariate and multivariate Cox regression analyses were utilized to confirm independent prognostic factors for OS and CSS. In age-stratified analysis (70–74; 75–79; 80–84; ≥ 85), the OS and CSS rates of patients in the surgery group were significantly higher than those of patients in the no-surgery group (all P < 0.001). Adjuvant therapy was an independent prognostic factor for OS and CSS in elderly patients with CRC (all P < 0.001). Further analysis showed that elderly colon cancer patients with stage III and stage IV disease gained a survival benefit from adjuvant chemotherapy. Adjuvant chemoradiotherapy can significantly improve OS and CSS in elderly rectal cancer patients with stage II, III, and IV disease. In conclusion, among CRC patients aged ≥ 70 years reported in the SEER database, treatment with surgical resection is significantly associated with improved OS and CSS. Moreover, adjuvant therapy led to a significant prognostic advantage for elderly advanced CRC patients who underwent surgery.

## Introduction

There are health-related consequences of the worldwide ageing population, which is growing^1^. This increase in population ageing is accompanied by a higher incidence of malignancies, one of which is colorectal cancer (CRC)^2^. Indeed, CRC is one of the most common malignancies of the digestive tract in Asia and most Western countries. According to GLOBOCAN 2020, CRC is the second most common cancer and has the second highest mortality rate for both sexes combined. It is estimated that approximately 1,930,283 people worldwide will develop the disease, of whom approximately 915,880 will die^3^. CRC is predominantly a disease of elderly individuals, with approximately 50% of patients being older than 70 years and more than 40% of patients being older than 75 years^4, 5^. Although no universal definition of “elderly” for CRC patients has been adopted, it is believed that it represents people aged 70–75 years and older^6–9^.

As surgery is the most effective therapeutic modality for CRC to date, primary treatment of CRC is surgical resection supplemented by chemotherapy-based strategies. Elderly patients are more prone to frailty, geriatric syndromes, comorbidities, polypharmacy, and a decline in organ function, and it is thus uncertain whether surgery and adjuvant therapy benefits these patients. Pereira et al. reported that BRAF mutations, which are associated with an aggressive clinical course and poor prognosis, are more frequent among CRC patients aged ≥ 75 years^10^. McCleary et al. demonstrated that tumour nuclear CTNNB1 expression is associated with higher mortality among elderly patients with CRC but not among younger patients^11^. Moreover, the life expectancy of elderly patients is relatively short. Thus, the treatment strategies concluded from cohorts of younger patients may not be completely applicable to elderly patients. What seems clear, though, is that the incidence of postoperative complications is much higher in elderly patients^12, 13^. Many studies have proven that postoperative complications after CRC resection are associated with decreased long-term survival, particularly severe postoperative complications^14, 15^, which may be one of the factors that influence the decisions of therapeutic strategies for elderly patients. As a result, more clinical studies focused on elderly patients should be performed to determine the optimal therapeutic strategy.

This study investigated the survival benefit of surgery and different therapeutic patterns for elderly CRC patients (i.e., ≥ 70 years old). Survival outcomes were retrospectively compared using propensity score matching (PSM) between patients who underwent surgery and those who did not. Subgroup analysis of prognosis was also performed for various ages and stages to explore impacts of different therapeutic patterns on CRC patients.

## Methods

### Patients and data

**Figure 1:**
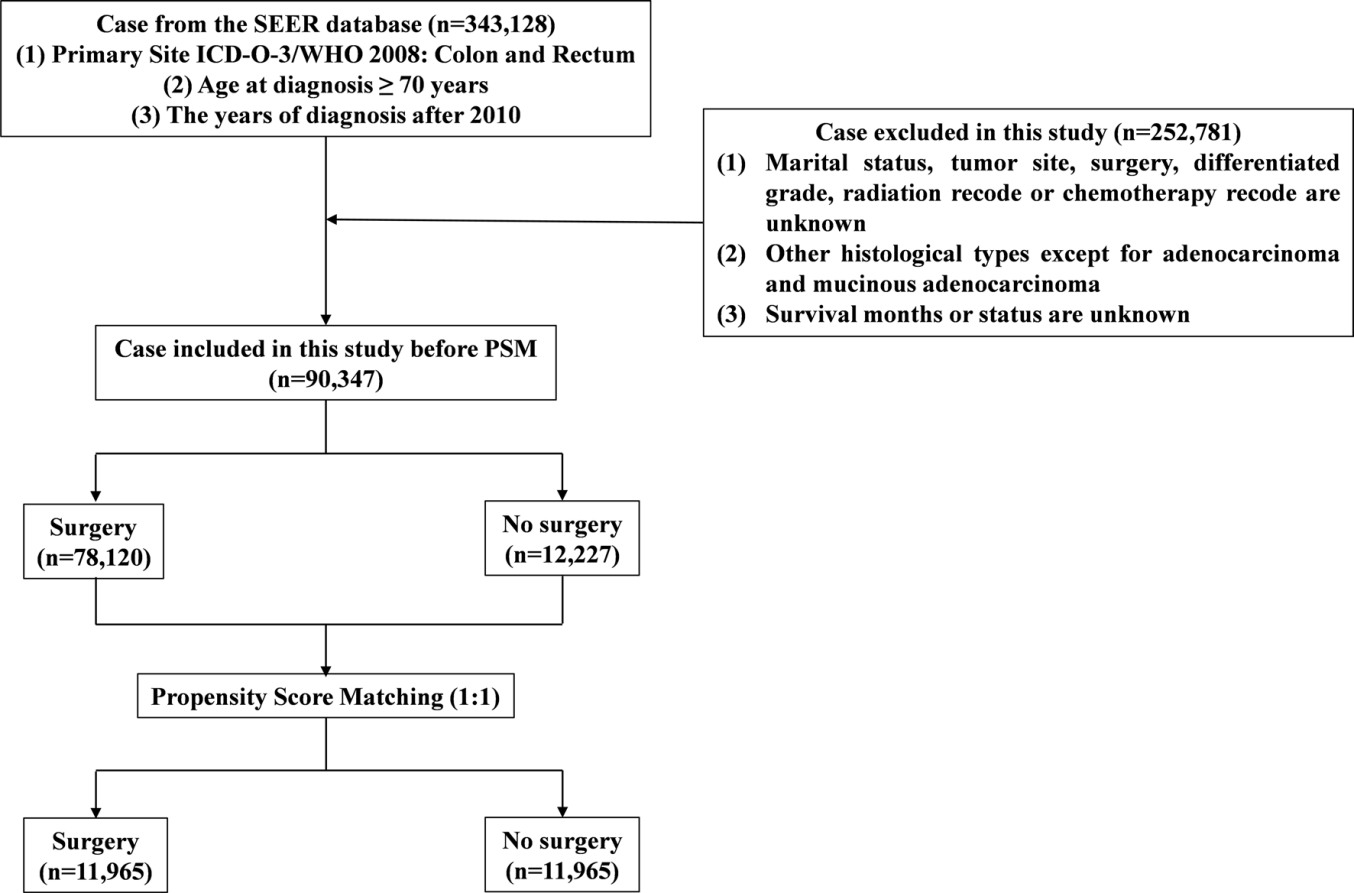
Flowchart for data filtration of older patients with colorectal cancer according to SEER dataset. PSM, propensity score matching.

Patient data were retrieved from the following Surveillance, Epidemiology, and End Results (SEER) database: Incidence-SEER Research Plus Data, 17 Registries, released in 2023 as a data source. SEER*Stat software was used to extract clinicopathologic and survival information. The inclusion criteria were as follows: (1) ({Site and Morphology. Site recode ICD-O-3/WHO 2008} = 'Colon and Rectum'); (2) ({Age at Diagnosis. Age recode with < 1 year olds} = '70–74 years', '75–79 years', '80–84 years', '85 + years'); (3) ({Race, Sex, Year Dx, Registry, County. Year of diagnosis} = '2010', '2011', '2012', '2013', '2014', '2015', '2016', '2017', '2018', '2019', '2020'). The exclusion criteria were as follows: (1) marital status, tumour site, surgery, differentiated grade, radiation recode or chemotherapy (CT) recode unknown; (2) histological types other than adenocarcinoma and mucinous adenocarcinoma; and (3) survival months or status unknown. A flow chart of the study population selection is displayed in Fig. 1.

All eligible patients were divided according to whether patients received surgery into a surgery group and a no-surgery group. No surgery was defined as no surgery of the primary site, only autopsy, or only excisional biopsy. Our study included all patients who met the inclusion and exclusion criteria to minimize selection bias. Propensity score analysis was used to control for potential confounding by the matching factors. Patients were propensity matched 1:1 into surgery and no-surgery groups through the nearest neighbour method with a calliper of 0.1 times the standard deviation of the propensity score. Variables used for matching were as follows: age, sex, year of diagnosis, race, marital status, tumour site, histological type, differentiated grade, radiotherapy (RT), and CT.

### Statistical analysis

All analyses were performed with IBM SPSS Statistics 25.0 software (IBM, Armonk, NY, USA) and R version 4.0.0 (http://www.r-project.org), and two-tailed P values < 0.05 were assessed as statistically significant. To balance covariance and reduce the bias of efficacy evaluation, we performed 1:1 PSM between the surgery group and no surgery group. Standardized differences were used to examine the balance across baseline covariates before and after matching, and a standardized difference below 10% was considered to be sufficiently reliable to provide well-balanced covariates after matching. Next, chi-square analysis was utilized to compare clinicopathologic characteristics in both matched and unmatched groups. A Cox regression model was applied for multivariate analysis to identify significant prognosticators. We calculated odds ratios (ORs) and 95% confidence intervals (CIs) for each prognosticator. The Kaplan–Meier method and log-rank test were used to compare overall survival (OS) and cancer-specific survival (CSS).

## Results

### Baseline characteristics

After screening, a total of 90,347 patients were included in the final analysis. Among them, 78,120 (86.47%) patients were enrolled in the surgery group and 12,227 (13.53%) in the no-surgery group. After ten characteristics were matched by PSM at a 1:1 ratio, 23,930 patients were included in our analysis. The baseline characteristics of the patients before and after PSM are summarized in Supplemental Table 1. We found significant differences between the groups regarding age, sex, year of diagnosis, race, marital status, tumour site, histological type, differentiated grade, RT, and CT among the clinicopathologic characteristics of the surgery and no-surgery groups before PSM. After matching by the propensity score, the baseline characteristics were similar for the matched cohort for most variables, except for age and CT. Estimates of standardized difference scores after matching were all less than 10%, which indicates that the baseline patient characteristics were balanced after matching (Supplementary Fig. 1).

### Survival outcomes of surgical patients and nonsurgical patients after PSM

**Figure 2:**
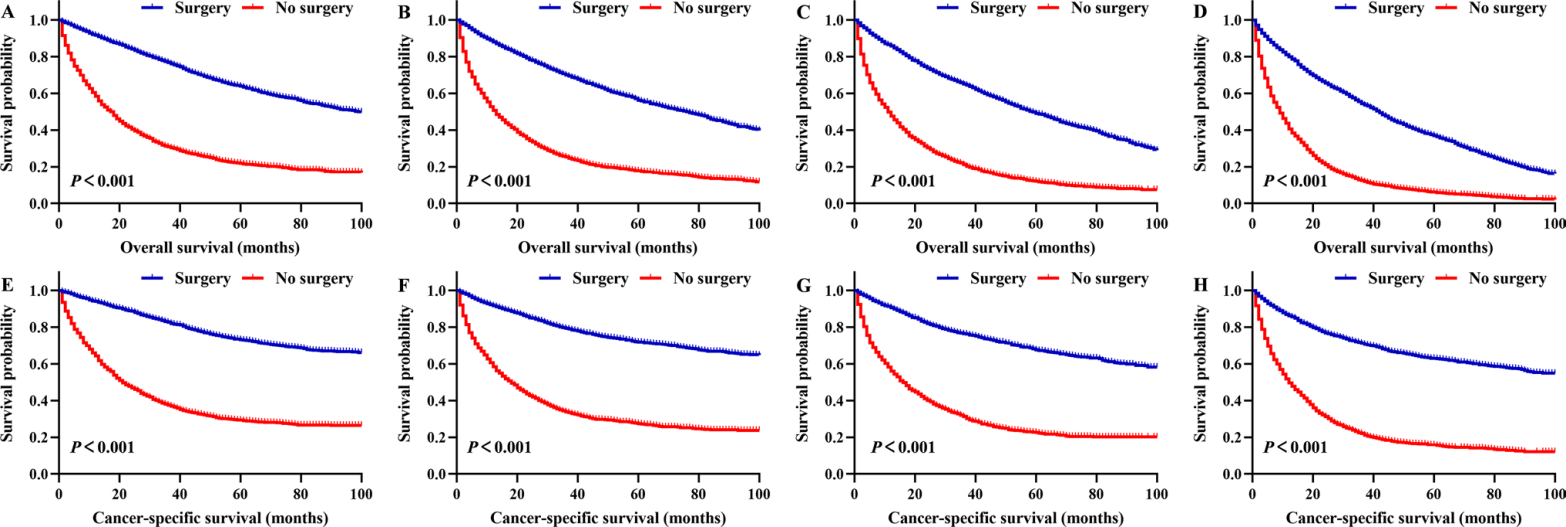
According to whether or not surgery, Kaplan–Meier survival analysis of colorectal cancer patients after propensity score matching. Overall survival for patients 70–74 years old (**A**), 75–79 years old (**B**), 80–84 years old (**C**), and 85+ years old (**D**) after PSM. Cancer-specific survival for patients 70–74 years old (**E**), 75–79 years old (**F**), 80–84 years old (**G**), and 85+ years old (**H**) after PSM. PSM, propensity score matching.

To compare the survival rate of the surgery and no-surgery groups at different ages, we divided the 23,930 CRC patients into four age stages (ages 70–74; 75–79; 80–84; ≥ 85): 5455, 5390, 5576, and 7509 patients aged 70–74, 75–79, 80–84, and ≥ 85, respectively. In CRC patients treated with surgery, the 5-year OS rates for those aged 70–74, 75–79, 80–84, and ≥ 85 years were 63.1%, 55.3%, 47.7%, and 35.2%, respectively, which were clearly better than those of patients not treated with surgery (Fig. 2A–D). Similarly, in CRC patients treated with surgery, the 5-year CSS rates for those aged 70–74, 75–79, 80–84, and ≥ 85 years were 72.6%, 70.9%, 66.4%, and 60.9%, respectively, significantly better prognoses than those of patients not treated with surgery (Fig. 2E–H). OS and CSS rates in CRC patients treated with surgery in each age group were significantly higher than those in the no-surgery group (all P < 0.001). Subgroup analysis according to tumour location was also performed. Overall, surgery, whether for colon or rectal cancer, can significantly improve prognosis (Supplementary Figs. 2, 3).

### Univariate and multivariate Cox analyses in patients after PSM

After PSM, multivariate Cox analysis showed that surgery (OR = 3.601, 95% CI 3.485–3.721, P < 0.001) was an independent predictive factor for OS (Supplemental Table 2). Analogously, the results of multivariate Cox analysis indicated that surgery (OR = 4.688, 95% CI 4.497–4.886, P < 0.001) correlated significantly with CSS (Supplemental Table 3). Remarkably, the OR of surgery was highest, and surgical treatment was the most significant protective factor for both OS and CSS in older patients with CRC. We also performed subgroup analysis by tumour location (Supplemental Tables 4–7). Similar results were also found for colon and rectal cancer analysed separately. TNM staging was not included in multivariate analysis because an accurate pathological stage could not be obtained for nonsurgical patients. Thus, we further compared the survival outcomes of surgical patients in different stages with nonsurgical patients. Compared with stage IV patients who underwent surgery, nonsurgical patients had a worse outcome (Supplementary Figs. 4, 5).

### Univariate and multivariate Cox analyses for patients who underwent surgery after PSM

Further univariate and multivariate analyses were performed for patients who underwent surgery after PSM. Due to the different prognoses and therapeutic patterns of colon and rectal cancer, we performed subgroup analysis based on tumour site. In patients with colon cancer, multivariate Cox analysis showed that CT (OR = 1.187, 95% CI 1.082–1.301, P < 0.001) was an independent predictive factor for OS (Supplemental Table 8). However, CT was not significantly associated with the CSS of colon cancer patients in multivariate Cox analysis (Supplemental Table 9).

In rectal cancer patients, multivariate Cox analysis indicated that RT (OR = 1.178, 95% CI 1.021–1.358, P = 0.024) and CT (OR = 1.306, 95% CI 1.127–1.512, P < 0.001) were independent predictive factors for OS (Supplemental Table 10). According to multivariate Cox regression analysis, RT (OR = 1.241, 95% CI 1.038–1.483, P = 0.018) remained an independent predictive factor for CSS in rectal cancer patients who underwent surgery. However, CT was not significantly associated with CSS in multivariate Cox analysis (Supplemental Table 11).

### Survival outcomes of different treatment patterns of patients who underwent surgery at different stages after PSM

**Figure 3:**
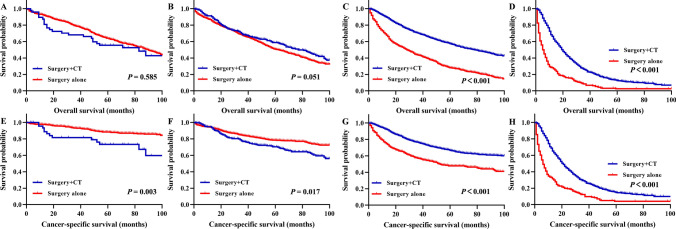
Kaplan–Meier survival analysis of colon cancer patients underwent surgery after propensity score matching (surgery + chemotherapy vs. surgery alone). Overall survival for patients with I stage (**A**), II stage (**B**), III stage (**C**), and IV stage (**D**). Cancer-specific survival for patients with I stage (**E**), II stage (**F**), III stage (**G**), and IV stage (**H**). CT, chemotherapy.

We conducted subgroup analysis stratified by stage to further analyse the influence of different treatment patterns on prognosis. In stage I colon cancer patients, the 5-year OS rates were 55.4% and 62.7% for patients with surgery + CT and surgery alone, respectively (P = 0.585, Fig. 3A); the 5-year CSS rates were 73.3% and 87.3% for patients with surgery + CT and surgery alone, respectively (P = 0.003, Fig. 3E). In patients with stage II disease, the 5-year OS rates were 58.8% and 48.1% for patients with surgery + CT and surgery alone, respectively (P = 0.051, Fig. 3B), and the 5-year CSS rates were 70.3% and 75.6% for patients with surgery + CT and surgery alone, respectively (P = 0.017, Fig. 3F). In patients treated with surgery + CT, the 5-year OS rates for patients with stage III and stage IV disease were 54.7% and 13.2%, respectively, which were significantly better prognoses than those of patients treated with surgery alone (all P < 0.001, Fig. 3C,D). Similarly, in patients treated with surgery + CT, the 5-year CSS rates for patients with stage III and stage IV disease were 66.7% and 14.8%, respectively, also significantly better prognoses than those of patients treated with surgery alone (all P < 0.001, Fig. 3G,H).

**Figure 4:**
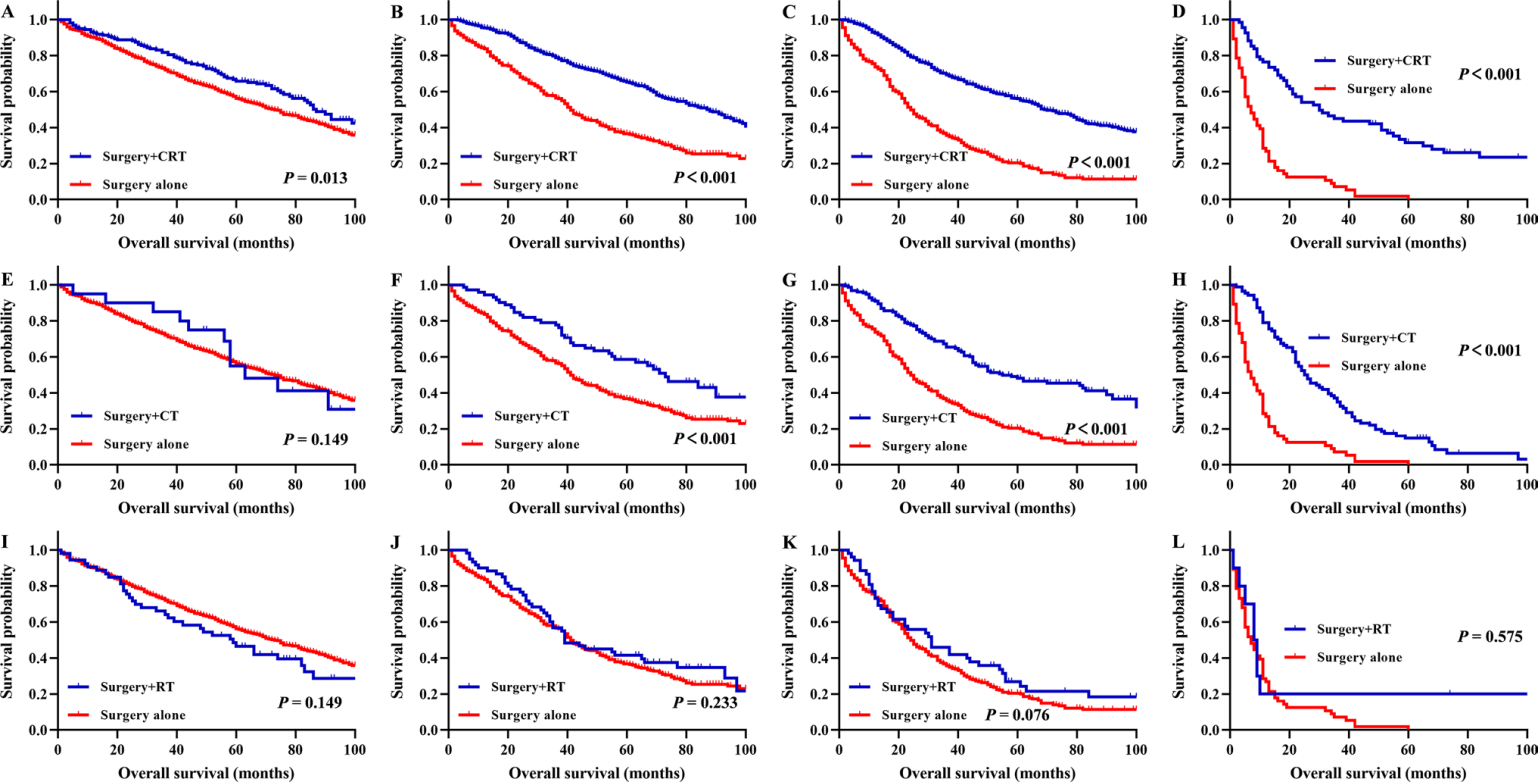
Survival analysis for overall survival of rectal cancer patients underwent surgery after propensity score matching. Overall survival for patients with I stage (**A**), II stage (**B**), III stage (**C**), and IV stage (**D**) (surgery + CRT vs. surgery alone). Overall survival for patients with I stage (**E**), II stage (**F**), III stage (**G**), and IV stage (**H**) (surgery + CT vs. surgery alone). Overall survival for patients with I stage (**I**), II stage (**J**), III stage (**K**), and IV stage (**L**) (surgery + RT vs. surgery alone). CRT, chemoradiotherapy; CT, chemotherapy; RT, radiotherapy.

**Figure 5:**
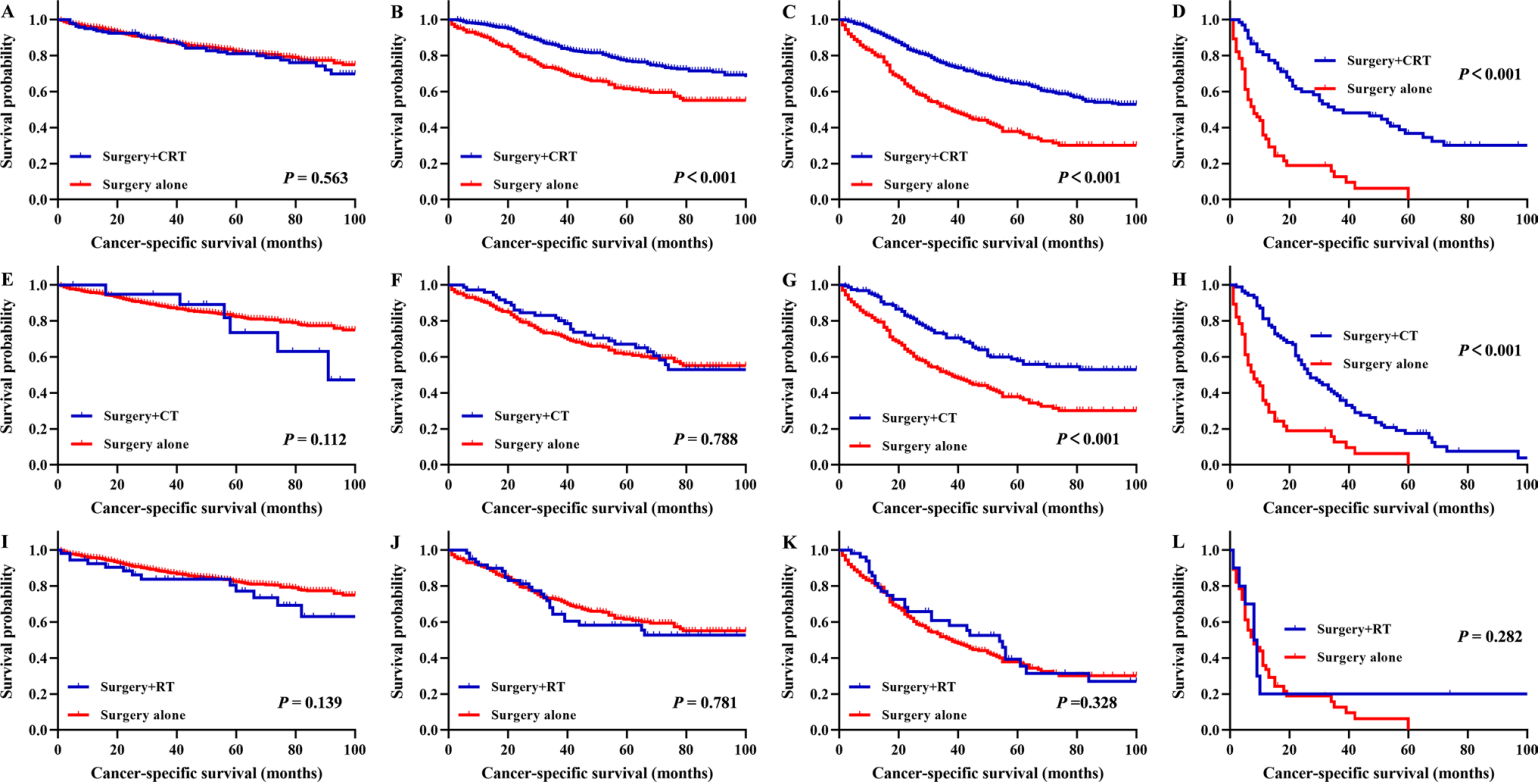
Survival analysis for cancer-specific survival of rectal cancer patients underwent surgery after propensity score matching. Cancer-specific survival for patients with I stage (**A**), II stage (**B**), III stage (**C**), and IV stage (**D**) (surgery + CRT vs. surgery alone). Cancer-specific survival for patients with I stage (**E**), II stage (**F**), III stage (**G**), and IV stage (**H**) (surgery + CT vs. surgery alone). Cancer-specific survival for patients with I stage (**I**), II stage (**J**), III stage (**K**), and IV stage (**L**) (surgery + RT vs. surgery alone). CRT, chemoradiotherapy; CT, chemotherapy; RT, radiotherapy.

In rectal cancer patients with stage I disease, the 5-year OS rates were 65.8% and 55.2% for patients with surgery + chemoradiotherapy (CRT) and surgery alone, respectively (P = 0.013, Fig. 4A), and the 5-year CSS rates were 81.0% and 82.0% for patients with surgery + CRT and surgery alone, respectively (P = 0.563, Fig. 5A). In patients treated with surgery + CRT, the 5-year OS rates for patients with stage II, stage III, and stage IV disease were 64.5%, 54.5% and 22.1%, respectively, which were significantly better prognoses than those of patients treated with surgery alone (all P < 0.001, Fig. 4B–D). Among patients treated with surgery + CRT, the 5-year CSS rates of stage II, stage III, and stage IV were 75.8%, 63.2%, and 26.8%, respectively, with significantly better prognosis than surgery alone (all P < 0.001, Fig. 5B–D).

In rectal cancer patients with stage I disease, the 5-year OS rates were 46.4% and 55.2% for patients with surgery + CT and surgery alone, respectively (P = 0.149, Fig. 4E); the 5-year CSS rates were 72.2% and 82.0% for patients with surgery + CT and surgery alone, respectively (P = 0.112, Fig. 5E). In patients with stage II disease, the 5-year OS rates were 60.3% and 38.1% for patients with surgery + CT and surgery alone, respectively (P < 0.001, Fig. 4F); the 5-year CSS rates were 63.3% and 61.6% for patients with surgery + CT and surgery alone, respectively (P = 0.788, Fig. 5F). In patients treated with surgery + CT, the 5-year OS rates for patients with stage III and stage IV disease were 51.5% and 16.3%, respectively, significantly better prognoses than those of patients treated with surgery alone (all P < 0.001, Fig. 4G,H). In patients treated with surgery + CT, the 5-year CSS rates for patients with stage III and stage IV disease were 60.3% and 18.2%, respectively, significantly better prognoses than those of patients treated with surgery alone (all P < 0.001, Fig. 5G,H).

In patients treated with surgery + RT, the 5-year OS rates for patients with stage I, II, III, and IV disease were 46.4%, 41.5%, 26.9% and 30.3%, respectively; these results indicated no significant improvement in prognosis compared with patients treated with surgery alone (all P > 0.05, Fig. 4I–L). Similarly, the 5-year CSS rates for patients treated with surgery + RT with stage I, II, III, and IV disease were 77.2%, 58.3%, 39.4% and 20.0%, respectively, which did not show a significant improvement in prognosis compared with patients treated with surgery alone (all P > 0.05, Fig. 5I–L).

### Survival outcomes of different treatment patterns of patients who underwent surgery in different age groups after PSM

**Figure 6:**
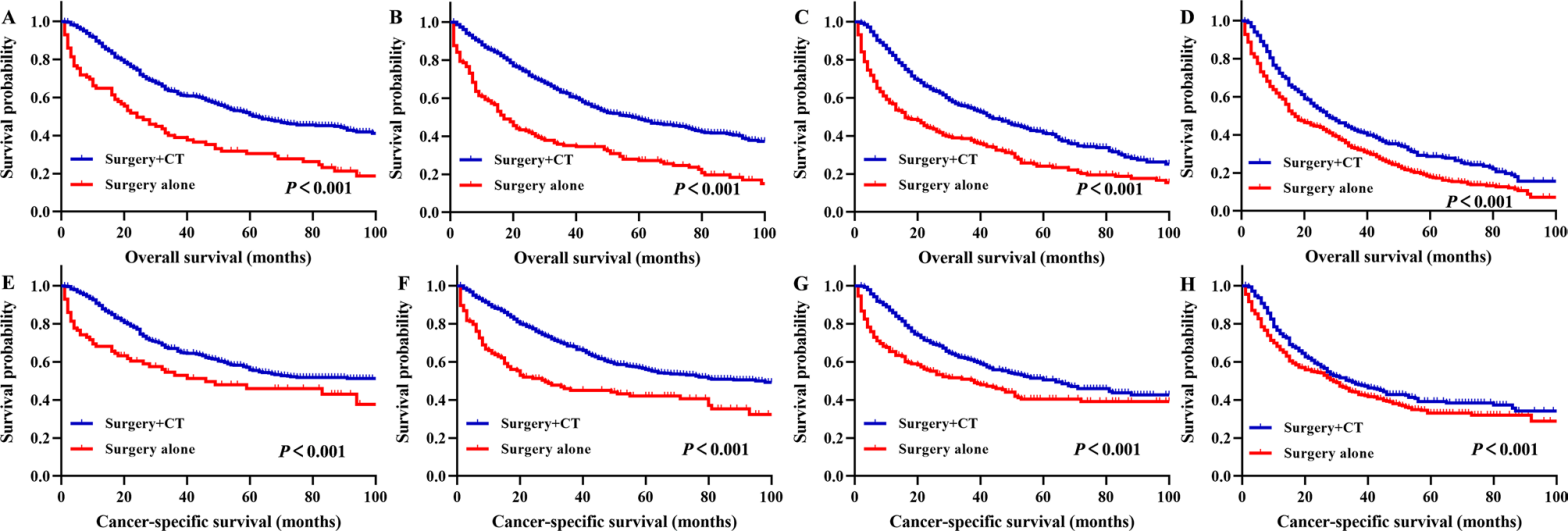
According to different treatment patterns, Kaplan–Meier survival analysis of colon cancer patients underwent surgery with III stage and IV stage in different age groups after propensity score matching. Overall survival for patients 70–74 years old (**A**), 75–79 years old (**B**), 80–84 years old (**C**), and 85+ years old (**D**). Cancer-specific survival for patients 70–74 years old (**E**), 75–79 years old (**F**), 80–84 years old (**G**), and 85+ years old (**H**). CT, chemotherapy.

Age-stratified analysis was also conducted to analyse the influence of different treatment patterns on prognosis. In stage III and stage IV colon cancer patients treated with surgery + CT, the 5-year OS rates for patients aged 70–74, 75–79, 80–84, and ≥ 85 years were 50.9%, 49.0%, 41.5%, and 28.7%, respectively; these rates were clearly better than those of patients treated with surgery alone (Fig. 6A–D). Similarly, in patients treated with surgery + CT, the 5-year CSS rates for patients aged 70–74, 75–79, 80–84, and ≥ 85 years were 55.9%, 55.7%, 50.6%, and 39.2%, respectively, which were significantly better prognoses than those of patients treated with surgery alone (Fig. 6E–H).

**Figure 7:**
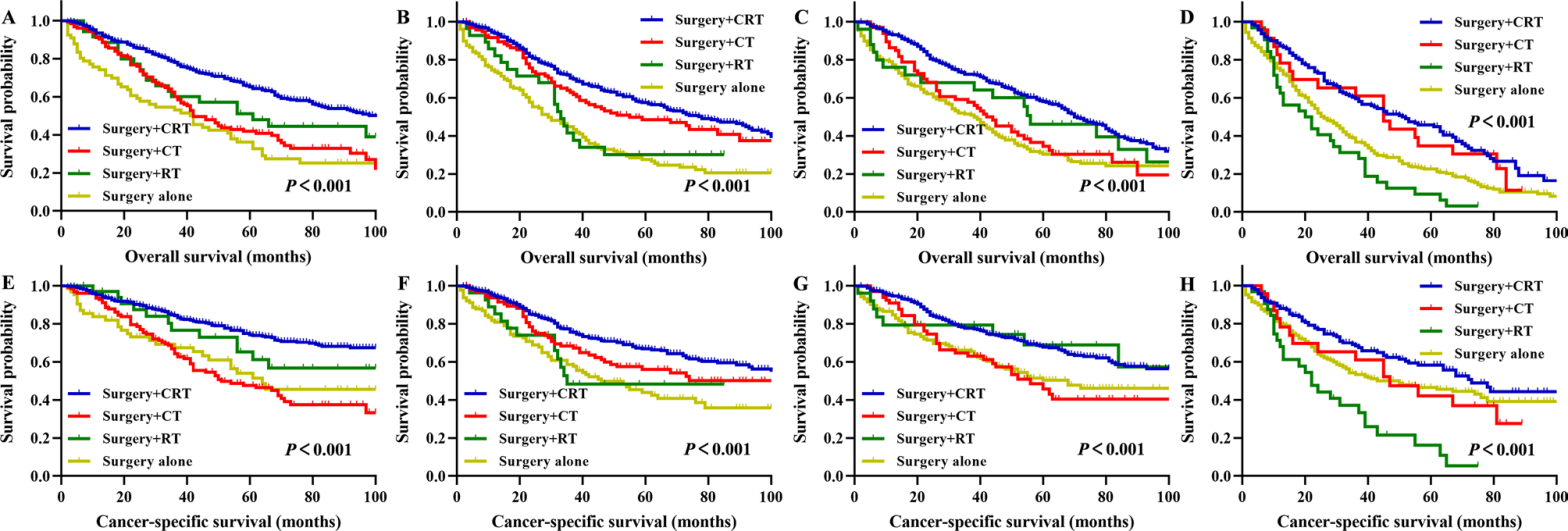
According to different treatment patterns, Kaplan–Meier survival analysis of rectal cancer patients underwent surgery with II stage, III stage and IV stage after propensity score matching. Overall survival for patients 70–74 years old (**A**), 75–79 years old (**B**), 80–84 years old (**C**), and 85+ years old (**D**). Cancer-specific survival for patients 70–74 years old (**E**), 75–79 years old (**F**), 80–84 years old (**G**), and 85+ years old (**H**). CRT, chemoradiotherapy; CT, chemotherapy; RT, radiotherapy.

In stage II, stage III, and stage IV rectal cancer patients treated with surgery + CRT, the 5-year OS rates for patients aged 70–74, 75–79, 80–84, and ≥ 85 years were 65.1%, 57.5%, 58.1%, and 45.8%, respectively, clearly better than those of patients treated with other treatment patterns (Fig. 7A–D). In patients treated with surgery + CRT, the 5-year CSS rates for patients aged 70–74, 75–79, 80–84, and ≥ 85 years were 75.0%, 66.7%, 67.8%, and 58.2%, respectively, significantly better prognoses than those of patients treated with other treatment patterns (Fig. 7E–H). The results of pairwise comparisons are displayed in Supplemental Table 12.

## Discussion

The incidence of CRC is known to increase with age. As life expectancy is increasing, the number of elderly CRC patients with decreased functional status and increased comorbidity is also expected to increase worldwide^2^. However, the relatively high risks of complications, postoperative sequelae, in-hospital mortality, and sensitivity to treatment toxicity in elderly patients with CRC have led to refusal of surgery and aggressive adjuvant treatments. In the present study, we carefully identified appropriate patients from the 17 SEER databases and investigated therapeutic patterns and survival outcomes. Our results showed that surgical resection was associated with significantly longer OS and CSS in elderly CRC patients regardless of how old the patients were. Furthermore, we found that surgery + CT was associated with an apparent survival benefit in elderly advanced colon cancer patients and that surgery + CRT could significantly improve prognosis in elderly advanced rectal cancer patients compared to other treatment patterns.

Surgery is widely believed to be the best approach for treating solid tumours^16^. However, decisions regarding whether surgery should be performed for elderly patients are sometimes difficult for clinicians, patients, and their families, as influenced by numerous factors. A retrospective multicentre study in Japan demonstrated that age was a factor that reduced the survival of patients with resected CRC (P < 0.001)^17^. Kocián et al. reported that compared with young patients, the poorer prognosis of elderly patients (≥ 75 years) is presumably due to their worse physiological reserve and higher incidence of comorbidities^18^. However, one systematic review concluded that the CSS of rectal cancer patients does not decrease with age^19^. Thus, age alone should not be a decisive factor in medical decisions. Additional information is likely needed for patients to decide whether to undergo surgery. To our knowledge, studies of whether elderly CRC patients should undergo surgical therapy have not yet been fully explored. In our research, PSM was conducted to balance baseline characteristics related to survival outcome. After matching, surgical therapy was an independent prognostic factor in multivariate Cox regression analysis, and further survival analysis showed that patients in the surgery group had better OS and CSS than those in the no-surgery group in different age groups. Therefore, surgical treatment may be prudently recommended for elderly CRC patients. Careful clinical observation and assessment of elderly patients, acceptable morbidity, anticipated life span, comorbidities, nutrition, and functional status should be considered when deciding surgical cure.

Adjuvant therapy to target occult metastasis is advocated for stage II cancer with high-risk features and stage III disease^20^. However, the standard adjuvant treatment regimens for elderly patients are currently unclear because patients aged ≥ 70 years are frequently excluded from randomized trials^21^. Moreover, some studies have reported that the clinical effectiveness of adjuvant CT in elderly CRC patients appears to be limited, with increased chemotherapy-related toxic side effects^22, 23^. Thus, it is necessary to analyse the efficacy of adjuvant therapy in elderly CRC patients, which is a special group of cancer patients. Sargent et al. combined data from seven randomized controlled trials (RCTs) and found that adjuvant CT had a significant positive effect on both OS and time to tumour recurrence for colon cancer and that age did not appear to affect this result^24^. In general, RCTs have strict eligibility inclusion and exclusion criteria and typically exclude elderly patients with comorbidities and high frailty^25^. Nevertheless, real-world studies, with less restrictive study populations, might be more reflective of the effectiveness of interventions in real settings. A systematic review of real-world studies suggested that adjuvant CT significantly improves OS but not disease-free survival (DFS) in elderly CRC patients^26^. Our research found that adjuvant CT was significantly associated with improved OS and CSS in stage III and stage IV colon cancer patients. This result converges with research derived from younger patients^27, 28^. However, our study was not able to demonstrate a prognostic benefit of adjuvant CT in elderly stage II colon cancer patients. This may be attributable to the inability to differentiate stage II patients into high- and low-risk groups. Many studies have demonstrated that risk factor stratification may contribute to efficient selection of stage II colon cancer patients who will benefit from adjuvant therapy^29, 30^. Our study also found that stage I patients who received CT had worse prognosis than those who did not. Stage I CRC patients have a very low risk of recurrence, and thus postoperative adjuvant therapy is not indicated. This phenomenon may be due to postoperative tumour recurrence or metastasis.

Neoadjuvant chemoradiotherapy (nCRT) can downstage locally advanced rectal cancer, which results in a lower rate of postoperative local recurrence and is now considered standard treatment for locally advanced rectal cancer worldwide^31^. Adjuvant CRT is currently offered routinely after radical resection for patients with rectal cancer receiving nCRT, though the role of adjuvant therapy in rectal cancer remains controversial. EORTC 22921 evaluated the effect of neoadjuvant and/or adjuvant CT on preoperative RT and radical resection^32^, with no significant impact of adjuvant CT on OS, DFS or the cumulative incidence of distant spread for the whole group. The Italian I-CNR-RT trial randomized 655 patients with locally advanced rectal cancer to 6 cycles of 5FU vs. observation after nCRT and surgery^33^. They also concluded no benefit for adjuvant CT in the 5-year OS, distant metastasis, and local recurrence. To our knowledge, there are no RCTs or other high-quality studies investigating the efficacy of nCRT and adjuvant CT in rectal cancer patients over 70 years. In our research, CRT was significantly associated with improved OS and CSS in stage II, stage III, and stage IV rectal cancer patients in all age groups. However, limited data prevented us from ascertaining whether CRT was administered before or after surgery in rectal cancer patients. Thus, future clinical trials should further explore the value of nCRT and adjuvant CRT in older adults with advanced rectal cancer.

According to recent data, the life expectancy of medically fit elderly patients is gradually increasing^34^. Therefore, a new diagnosis of older adult CRC patient has the potential to increase life expectancy if treated. Regardless, the physiological heterogeneity of elderly patients with frequent discrepancies between biological and chronological age is coupled with additional complications. The International Society of Geriatric Oncology recommends that elderly CRC patients undergo preoperative evaluation of biological age based on the physiological side effects of ageing, physical and mental ability, and social support^35^. Thus, it is crucial for clinicians to distinguish fit from more vulnerable older patients. Moreover, outcomes related to surgery and adjuvant therapy should be well considered, such as postoperative morbidity and mortality, functional status, quality of life, toxicity of therapy, completion of therapy, and survival. A comprehensive geriatric assessment system should be established to aid in such therapeutic decisions. After adequate evaluation, treatment can be adjusted to maximize effectiveness and better meet the individual requirements of older patients. As a systematic review of seven existing frailty screening methods in older patients receiving surgery, RT, or CT concluded insufficient discriminative power to improve patient selection^36^, further research is required to establish a more efficient geriatric biological age evaluation system in oncology that can evaluate benefit/risk ratios of various treatment interventions for patients^37^.

Although the SEER database provides public data to investigate such clinical problems, there are several limitations in this study. First, treatment information, including RT dosing, CT regimen, and the sequence of CT, was not recorded in the SEER database, and we did not analyse the role of these factors. In addition, SEER records did not include information about locoregional relapse or distant metastasis; thus, we were unable to assess local recurrence survival and DFS. Further research directions include appropriate CT regimens used to treat elderly CRC patients, the effect of postoperative adjuvant therapy on elderly rectal cancer patients who receive neoadjuvant therapy, and screening appropriate elderly CRC patients who will benefit from adjuvant therapy.

## Conclusion

The current study indicates that surgical resection is significantly associated with improved prognosis for CRC patients aged ≥ 70 years in 17 SEER databases. Moreover, adjuvant therapy showed a significant prognostic advantage for elderly advanced CRC patients who underwent surgery in all age groups. We look forward to future high-quality prospective clinical studies that can reliably validate our conclusions.

### Supplementary Information

Supplementary Tables.

Supplementary Figures.

## Data Availability

The data that support the findings of this study are available from the corresponding author upon reasonable request.

## Abbreviations

CRC: Colorectal cancer
PSM: Propensity score matching
SEER: The Surveillance, Epidemiology, and End Results
CRT: Chemoradiotherapy
CT: Chemotherapy
RT: Radiotherapy
OR: Odds ratio
CIs: Confidence intervals
OS: Overall survival
CSS: Cancer-specific survival
nCRT: Neoadjuvant chemoradiotherapy
RCTs: Randomized controlled trials
DFS: Disease-free survival
