# Supplementary material for: Real-world analysis of survival benefit of surgery and adjuvant therapy in elderly patients with colorectal cancer
Source: Sci Rep. 2023 Sep 8;13:14866. doi: 10.1038/s41598-023-41713-1 (PMC10491681; doi:10.1038/s41598-023-41713-1)
Supplement: Supplementary file 1 — Supplementary Tables. [file 41598_2023_41713_MOESM1_ESM.docx]

**Real-world analysis of survival benefit of surgery and adjuvant therapy in elderly patients with colorectal cancer**

Zhang et al.

(Supplementary tables)

**Results**

**Baseline characteristics**

**Supplementary Table 1. Baseline characteristics of all eligible patients before and after propensity score matching.**

|  | | Before matching | | *p*-value | After matching | | | *p*-value |
| --- | --- | --- | --- | --- | --- | --- | --- | --- |
|  |  | Surgery  (n=78120) | No surgery  (n=12227) |  | Surgery  (n=11965) | No surgery  (n=11965) | |  |
| Age, No.(%) | |  |  | ＜0.001 |  |  | | 0.022 |
|  | 70-74 years | 22445 (28.7%) | 2714 (22.2%) |  | 2741 (22.9%) | | 2714 (22.7%) |  |
|  | 75-79 years | 20356 (26.1%) | 2621 (21.4%) |  | 2772 (23.2%) | | 2618 (21.9%) |  |
|  | 80-84 years | 17961 (23.0%) | 2850 (23.3%) |  | 2795 (23.4%) | | 2781 (23.2%) |  |
|  | 85+ years | 17358 (22.2%) | 4042 (33.1%) |  | 3657 (30.6%) | | 3852 (32.2%) |  |
| Sex, No.(%) | |  |  | ＜0.001 |  |  | | 0.365 |
|  | Male | 37105 (47.5%) | 6290 (51.4%) |  | 6203 (51.8%) | | 6133 (51.3%) |  |
|  | Female | 41015 (52.5%) | 5937 (48.6%) |  | 5762 (48.2%) | | 5832 (48.7%) |  |
| Year of diagnosis, No.(%) | |  |  | ＜0.001 |  |  | | 0.960 |
|  | 2010 | 10764 (13.8%) | 1448 (11.8%) |  | 1414 (11.8%) | | 1438 (12.0%) |  |
|  | 2011 | 10375 (13.3%) | 1463 (12.0%) |  | 1442 (12.1%) | | 1454 (12.2%) |  |
|  | 2012 | 10089 (12.9%) | 1471 (12.0%) |  | 1443 (12.1%) | | 1456 (12.2%) |  |
|  | 2013 | 9767 (12.5%) | 1457 (11.9%) |  | 1444 (12.1%) | | 1428 (11.9%) |  |
|  | 2014 | 9490 (12.1%) | 1609 (13.2%) |  | 1538 (12.9%) | | 1578 (13.2%) |  |
|  | 2015 | 9320 (11.9%) | 1553 (12.7%) |  | 1531 (12.8%) | | 1518 (12.7%) |  |
|  | 2016 | 9303 (11.9%) | 1552 (12.7%) |  | 1546 (12.9%) | | 1490 (12.5%) |  |
|  | 2017 | 9012 (11.5%) | 1674 (13.7%) |  | 1607 (13.4%) | | 1603 (13.4%) |  |
| Race, No.(%) | |  |  | ＜0.001 |  |  | | 0.219 |
|  | White | 65168 (83.4%) | 9915 (81.1%) |  | 9624 (80.4%) | | 9714 (81.2%) |  |
|  | Black | 6309 (8.1%) | 1184 (9.7%) |  | 1164 (9.7%) | | 1150 (9.6%) |  |
|  | Others^＊^ | 6643 (8.5%) | 1128 (9.2%) |  | 1177 (9.8%) | | 1101 (9.2%) |  |
| Marital status, No.(%) | |  |  | ＜0.001 |  |  | | 0.917 |
|  | Married | 39915 (51.1%) | 5420 (44.3%) |  | 5363 (44.8%) | | 5371 (44.9%) |  |
|  | Single | 38205 (48.9%) | 6807 (55.7%) |  | 6602 (55.2%) | | 6594 (55.1%) |  |
| Tumor site, No.(%) | |  |  | ＜0.001 |  |  | | 0.723 |
|  | Colon cancer | 69553 (89.0%) | 7030 (57.5%) |  | 7057 (59.0%) | | 7030 (58.8%) |  |
|  | Rectal cancer | 8567 (11.0%) | 5197 (42.5%) |  | 4908 (41.0%) | | 4935 (41.2%) |  |
| Grade, No.(%) | |  |  | ＜0.001 |  |  | | 0.122 |
|  | Well differentiated (Grade I) | 7259 (9.3%) | 1313 (10.7%) |  | 1267 (10.6%) | | 1267 (10.6%) |  |
|  | Moderately differentiated (Grade II) | 55026 (70.4%) | 8627 (70.6%) |  | 8551 (71.5%) | | 8435 (70.5%) |  |
|  | Poorly differentiated (Grade III) | 12986 (16.6%) | 2103 (17.2%) |  | 1947 (16.3%) | | 2079 (17.4%) |  |
|  | Undifferentiated (Grade IV) | 2849 (3.6%) | 184 (1.5%) |  | 200 (1.7%) | | 184 (1.5%) |  |
| Histology, No.(%) | |  |  | ＜0.001 |  |  | | 0.240 |
|  | Adenocarcinoma | 70270 (90.0%) | 11748 (96.1%) |  | 11521 (96.3%) | | 11486 (96.0%) |  |
|  | Mucinous adenocarcinoma | 7850 (10.0%) | 479 (3.9%) |  | 444 (3.7%) | | 479 (4.0%) |  |
| Radiotherapy, No.(%) | |  |  | ＜0.001 |  |  | | 0.404 |
|  | Yes | 4949 (6.3%) | 2662 (21.8%) |  | 2408 (20.1%) | | 2460 (20.6%) |  |
|  | None/Unknown | 73171 (93.7%) | 9565 (78.2%) |  | 9557 (79.9%) | | 9505 (79.4%) |  |
| Chemotherapy, No.(%) | |  |  | ＜0.001 |  |  | | ＜0.001 |
|  | Yes | 18376 (23.5%) | 4225 (34.6%) |  | 4295 (35.9%) | | 4012 (33.5%) |  |
|  | None/Unknown | 59744 (76.5%) | 8002 (65.4%) |  | 7670 (64.1%) | | 7953 (66.5%) |  |
| ^＊^Asian, Pacific Islander, American Indian or Alaska Native | | | | | | | | |

**Univariate and multivariate Cox analyses** **in patients after PSM**

**Supplemental Table 2. Univariate and multivariate analysis of overall survival for patients after propensity score matching.**

| **Variables** | | **Univariate analysis** | |  | **Multivariate analysis** | |
| --- | --- | --- | --- | --- | --- | --- |
|  |  | **Odds ratio (95% CI)** | ***p*-value** |  | **Odds ratio (95% CI)** | ***p*-value** |
| Age | |  | ＜0.001 |  |  | ＜0.001 |
|  | 70-74 years | Reference |  |  | Reference |  |
|  | 75-79 years | 1.180 (1.124-1.238) |  |  | 1.212 (1.155-1.272) |  |
|  | 80-84 years | 1.421 (1.356-1.489) |  |  | 1.456 (1.389-1.526) |  |
|  | 85+ years | 1.869 (1.790-1.951) |  |  | 1.781 (1.701-1.864) |  |
| Sex | |  | 0.576 |  |  |  |
|  | Male | Reference |  |  |  |  |
|  | Female | 0.991 (0.962-1.022) |  |  |  |  |
| Year of diagnosis | |  | 0.064 |  |  |  |
|  | 2010-2013 | Reference |  |  |  |  |
|  | 2014-2017 | 0.971 (0.941-1.002) |  |  |  |  |
| Race | |  | ＜0.001 |  |  | ＜0.001 |
|  | White | Reference |  |  | Reference |  |
|  | Black | 1.087 (1.033-1.143) |  |  | 1.108 (1.053-1.167) |  |
|  | Others^＊^ | 0.875 (0.829-0.923) |  |  | 0.900 (0.853-0.950) |  |
| Marital status | |  | ＜0.001 |  |  | ＜0.001 |
|  | Married | Reference |  |  | Reference |  |
|  | Single | 1.206 (1.170-1.244) |  |  | 1.112 (1.077-1.147) |  |
| Tumor site | |  | ＜0.001 |  |  | ＜0.001 |
|  | Rectal cancer | Reference |  |  | Reference |  |
|  | Colon cancer | 1.362 (1.320-1.405) |  |  | 1.306 (1.260-1.353) |  |
| Grade | |  | ＜0.001 |  |  | ＜0.001 |
|  | Well differentiated (Grade I) | Reference |  |  | Reference |  |
|  | Moderately differentiated (Grade II) | 1.235 (1.171-1.302) |  |  | 1.310 (1.242-1.382) |  |
|  | Poorly differentiated (Grade III) | 1.768 (1.664-1.879) |  |  | 1.938 (1.822-2.062) |  |
|  | Undifferentiated (Grade IV) | 1.810 (1.601-2.046) |  |  | 2.068 (1.828-2.340) |  |
| Histology | |  | 0.401 |  |  |  |
|  | Adenocarcinoma | Reference |  |  |  |  |
|  | Mucinous adenocarcinoma | 0.975 (0.920-1.034) |  |  |  |  |
| Surgery | |  | ＜0.001 |  |  | ＜0.001 |
|  | Yes | Reference |  |  | Reference |  |
|  | No | 3.223 (3.121-3.327) |  |  | 3.601 (3.485-3.721) |  |
| Radiotherapy | |  | ＜0.001 |  |  | ＜0.001 |
|  | Yes | Reference |  |  | Reference |  |
|  | None/Unknown | 1.420 (1.365-1.477) |  |  | 1.269 (1.208-1.334) |  |
| Chemotherapy | |  | ＜0.001 |  |  | 0.010 |
|  | Yes | Reference |  |  | Reference |  |
|  | None/Unknown | 1.317 (1.275-1.361) |  |  | 1.052 (1.012-1.094) |  |
| ^＊^Asian, Pacific Islander, American Indian or Alaska Native | | | | | | |

**Supplemental Table 3. Univariate and multivariate analysis of cancer-specific survival for patients after propensity score matching.**

| **Variables** | | **Univariate analysis** | |  | **Multivariate analysis** | |
| --- | --- | --- | --- | --- | --- | --- |
|  |  | **Odds ratio (95% CI)** | ***p*-value** |  | **Odds ratio (95% CI)** | ***p*-value** |
| Age | |  | ＜0.001 |  |  | ＜0.001 |
|  | 70-74 years | Reference |  |  | Reference |  |
|  | 75-79 years | 1.068 (1.009-1.130) |  |  | 1.123 (1.061-1.189) |  |
|  | 80-84 years | 1.226 (1.160-1.295) |  |  | 1.300 (1.230-1.375) |  |
|  | 85+ years | 1.508 (1.433-1.586) |  |  | 1.543 (1.461-1.629) |  |
| Sex | |  | 0.006 |  |  | ＜0.001 |
|  | Male | Reference |  |  | Reference |  |
|  | Female | 1.052 (1.014-1.092) |  |  | 0.925 (0.889-0.963) |  |
| Year of diagnosis | |  | 0.927 |  |  |  |
|  | 2010-2013 | Reference |  |  |  |  |
|  | 2014-2017 | 0.998 (0.962-1.036) |  |  |  |  |
| Race | |  | ＜0.001 |  |  | 0.001 |
|  | White | Reference |  |  | Reference |  |
|  | Black | 1.093 (1.028-1.162) |  |  | 1.106 (1.040-1.177) |  |
|  | Others^＊^ | 0.914 (0.857-0.974) |  |  | 0.954 (0.894-1.018) |  |
| Marital status | |  | ＜0.001 |  |  | ＜0.001 |
|  | Married | Reference |  |  | Reference |  |
|  | Single | 1.186 (1.142-1.230) |  |  | 1.181 (1.134-1.230) |  |
| Tumor site | |  | ＜0.001 |  |  | ＜0.001 |
|  | Rectal cancer | Reference |  |  | Reference |  |
|  | Colon cancer | 1.392 (1.341-1.446) |  |  | 1.376 (1.317-1.438) |  |
| Grade | |  | ＜0.001 |  |  | ＜0.001 |
|  | Well differentiated (Grade I) | Reference |  |  | Reference |  |
|  | Moderately differentiated (Grade II) | 1.401 (1.308-1.501) |  |  | 1.453 (1.356-1.557) |  |
|  | Poorly differentiated (Grade III) | 2.202 (2.040-2.377) |  |  | 2.365 (2.189-2.556) |  |
|  | Undifferentiated (Grade IV) | 2.145 (1.851-2.485) |  |  | 2.426 (2.092-2.813) |  |
| Histology | |  | 0.319 |  |  |  |
|  | Adenocarcinoma | Reference |  |  |  |  |
|  | Mucinous adenocarcinoma | 0.965 (0.900-1.035) |  |  |  |  |
| Surgery | |  | ＜0.001 |  |  | ＜0.001 |
|  | Yes | Reference |  |  | Reference |  |
|  | No | 4.207 (4.040-4.382) |  |  | 4.688(4.497-4.886) |  |
| Radiotherapy | |  | ＜0.001 |  |  | ＜0.001 |
|  | Yes | Reference |  |  | Reference |  |
|  | None/Unknown | 1.350 (1.288-1.415) |  |  | 1.360 (1.282-1.442) |  |
| Chemotherapy | |  | 0.023 |  |  | ＜0.001 |
|  | Yes | Reference |  |  | Reference |  |
|  | None/Unknown | 1.045 (1.006-1.085) |  |  | 0.851 (0.814-0.890) |  |
| ^＊^Asian, Pacific Islander, American Indian or Alaska Native | | | | | | |

**Univariate and multivariate Cox analyses in patients after PSM according to tumor location**

In colon cancer patients, multivariate Cox analysis showed that surgery (OR = 3.927, 95%CI: 3.751-4.110, P < 0.001) was the independent predictive factors for OS (**Supplementary Table 4**). Analogously, surgery (OR = 4.741, 95%CI: 4.489-5.008, P < 0.001) was the independent predictive factors for CSS (**Supplementary Table 5**).

In rectal cancer patients, multivariate Cox analysis showed that surgery (OR = 3.925, 95%CI: 3.749-4.108, P < 0.001) was independent risk factors for the OS of the patients (**Supplementary Table 6**). Analogously, surgery (OR = 4.740, 95%CI: 4.487-5.006, P < 0.001) was the independent predictive factors for CSS (**Supplementary Table 7**).

**Supplementary Table 4. Univariate and multivariate analysis of overall survival for colon patients after propensity score matching.**

| **Variables** | | **Univariate analysis** | |  | **Multivariate analysis** | |
| --- | --- | --- | --- | --- | --- | --- |
|  |  | **Odds ratio (95% CI)** | ***p*-value** |  | **Odds ratio (95% CI)** | ***p*-value** |
| Age | |  | ＜0.001 |  |  | ＜0.001 |
|  | 70-74 years | Reference |  |  | Reference |  |
|  | 75-79 years | 1.019 (0.959-1.084) |  |  | 1.099 (1.033-1.169) |  |
|  | 80-84 years | 1.325 (1.247-1.407) |  |  | 1.316 (1.238-1.400) |  |
|  | 85+ years | 1.604 (1.519-1.694) |  |  | 1.542 (1.454-1.636) |  |
| Sex | |  | ＜0.001 |  |  | ＜0.001 |
|  | Male | Reference |  |  | Reference |  |
|  | Female | 0.864 (0.831-0.898) |  |  | 0.851 (0.817-0.887) |  |
| Year of diagnosis | |  | 0.196 |  |  |  |
|  | 2010-2013 | Reference |  |  |  |  |
|  | 2014-2017 | 1.026 (0.987-1.068) |  |  |  |  |
| Race | |  | ＜0.001 |  |  | ＜0.001 |
|  | White | Reference |  |  | Reference |  |
|  | Black | 0.723 (0.686-0.763) |  |  | 1.097 (1.037-1.161) |  |
|  | Others^＊^ | 0.625 (0.588-0.665) |  |  | 0.880 (0.826-0.938) |  |
| Marital status | |  | ＜0.001 |  |  | ＜0.001 |
|  | Married | Reference |  |  | Reference |  |
|  | Single | 1.107 (1.064-1.151) |  |  | 1.138 (1.091-1.197) |  |
| Grade | |  | ＜0.001 |  |  | ＜0.001 |
|  | Well differentiated (Grade I) | Reference |  |  | Reference |  |
|  | Moderately differentiated (Grade II) | 1.399 (1.262-1.551) |  |  | 1.235 (1.159-1.315) |  |
|  | Poorly differentiated (Grade III) | 1.579 (1.484-1.679) |  |  | 1.802 (1.681-1.932) |  |
|  | Undifferentiated (Grade IV) | 1.795 (1.675-1.923) |  |  | 1.890 (1.703-2.098) |  |
| Histology | |  | ＜0.001 |  |  |  |
|  | Adenocarcinoma | Reference |  |  |  |  |
|  | Mucinous adenocarcinoma | 0.834 (0.779-0.894) |  |  |  |  |
| Surgery | |  | ＜0.001 |  |  | ＜0.001 |
|  | Yes | Reference |  |  | Reference |  |
|  | No | 3.731 (3.580-3.888) |  |  | 3.927 (3.751-4.110) |  |
| Chemotherapy | |  | ＜0.001 |  |  | ＜0.001 |
|  | Yes | Reference |  |  | Reference |  |
|  | None/Unknown | 1.454 (1.395-1.515) |  |  | 1.147 (1.097-1.200) |  |
| ^＊^Asian, Pacific Islander, American Indian or Alaska Native | | | | | | |

**Supplementary Table 5. Univariate and multivariate analysis of cancer-specific survival for colon patients after propensity score matching.**

| **Variables** | | **Univariate analysis** | |  | **Multivariate analysis** | |
| --- | --- | --- | --- | --- | --- | --- |
|  |  | **Odds ratio (95% CI)** | ***p*-value** |  | **Odds ratio (95% CI)** | ***p*-value** |
| Age | |  | ＜0.001 |  |  | ＜0.001 |
|  | 70-74 years | Reference |  |  | Reference |  |
|  | 75-79 years | 0.949 (0.885-1.018) |  |  | 1.056 (0.984-1.133) |  |
|  | 80-84 years | 1.131 (1.055-1.213) |  |  | 1.173 (1.093-1.259) |  |
|  | 85+ years | 1.310 (1.231-1.395) |  |  | 1.369 (1.278-1.466) |  |
| Sex | |  | ＜0.001 |  |  | ＜0.001 |
|  | Male | Reference |  |  | Reference |  |
|  | Female | 0.875 (0.837-0.916) |  |  | 0.912 (0.869-0.958) |  |
| Year of diagnosis | |  | 0.066 |  |  |  |
|  | 2010-2013 | Reference |  |  |  |  |
|  | 2014-2017 | 1.044 (0.997-1.093) |  |  |  |  |
| Race | |  | ＜0.001 |  |  | ＜0.001 |
|  | White | Reference |  |  | Reference |  |
|  | Black | 0.703 (0.659-0.749) |  |  | 1.106 (1.035-1.182) |  |
|  | Others^＊^ | 0.592 (0.549-0.638) |  |  | 0.912 (0.869-0.956) |  |
| Marital status | |  | 0.007 |  |  | ＜0.001 |
|  | Married | Reference |  |  | Reference |  |
|  | Single | 1.065 (1.017-1.115) |  |  | 1.128 (1.074-1.186) |  |
| Grade | |  | ＜0.001 |  |  | ＜0.001 |
|  | Well differentiated (Grade I) | Reference |  |  | Reference |  |
|  | Moderately differentiated (Grade II) | 1.680 (1.482-1.905) |  |  | 1.409 (1.299-1.527) |  |
|  | Poorly differentiated (Grade III) | 1.934 (1.786-2.094) |  |  | 2.220 (2.034-2.423) |  |
|  | Undifferentiated (Grade IV) | 2.276 (2.087-2.483) |  |  | 2.347 (2.066-2.666) |  |
| Histology | |  | ＜0.001 |  |  |  |
|  | Adenocarcinoma | Reference |  |  |  |  |
|  | Mucinous adenocarcinoma | 0.795 (0.732-0.865) |  |  |  |  |
| Surgery | |  | ＜0.001 |  |  | ＜0.001 |
|  | Yes | Reference |  |  | Reference |  |
|  | No | 4.328 (4.117-4.549) |  |  | 4.741 (4.489-5.008) |  |
| Chemotherapy | |  | ＜0.001 |  |  | 0.001 |
|  | Yes | Reference |  |  | Reference |  |
|  | None/Unknown | 1.142 (1.089-1.196) |  |  | 0.917 (0.871-0.966) |  |
| ^＊^Asian, Pacific Islander, American Indian or Alaska Native | | | | | | |

**Supplementary Table 6. Univariate and multivariate analysis of overall survival for rectal patients after propensity score matching.**

| **Variables** | | **Univariate analysis** | |  | **Multivariate analysis** | |
| --- | --- | --- | --- | --- | --- | --- |
|  |  | **Odds ratio (95% CI)** | ***p*-value** |  | **Odds ratio (95% CI)** | ***p*-value** |
| Age | |  | ＜0.001 |  |  | ＜0.001 |
|  | 70-74 years | Reference |  |  | Reference |  |
|  | 75-79 years | 1.019 (0.959-1.084) |  |  | 1.098 (1.032-1.168) |  |
|  | 80-84 years | 1.325 (1.247-1.407) |  |  | 1.319 (1.240-1.402) |  |
|  | 85+ years | 1.604 (1.519-1.694) |  |  | 1.543 (1.454-1.637) |  |
| Sex | |  | ＜0.001 |  |  | ＜0.001 |
|  | Male | Reference |  |  | Reference |  |
|  | Female | 0.864 (0.831-0.898) |  |  | 0.850 (0.815-0.885) |  |
| Year of diagnosis | |  | 0.196 |  |  |  |
|  | 2010-2013 | Reference |  |  |  |  |
|  | 2014-2017 | 1.026 (0.987-1.068) |  |  |  |  |
| Race | |  | ＜0.001 |  |  | ＜0.001 |
|  | White | Reference |  |  | Reference |  |
|  | Black | 0.723 (0.686-0.763) |  |  | 1.093 (1.033-1.156) |  |
|  | Others^＊^ | 0.625 (0.588-0.665) |  |  | 0.877 (0.823-0.935) |  |
| Marital status | |  | ＜0.001 |  |  | ＜0.001 |
|  | Married | Reference |  |  | Reference |  |
|  | Single | 1.107 (1.064-1.151) |  |  | 1.139 (1.092-1.187) |  |
| Grade | |  | ＜0.001 |  |  | ＜0.001 |
|  | Well differentiated (Grade I) | Reference |  |  | Reference |  |
|  | Moderately differentiated (Grade II) | 1.399 (1.262-1.551) |  |  | 1.237 (1.162-1.318) |  |
|  | Poorly differentiated (Grade III) | 1.579 (1.484-1.679) |  |  | 1.801 (1.680-1.931) |  |
|  | Undifferentiated (Grade IV) | 1.795 (1.675-1.923) |  |  | 1.887 (1.700-2.094) |  |
| Histology | |  | ＜0.001 |  |  |  |
|  | Adenocarcinoma | Reference |  |  |  |  |
|  | Mucinous adenocarcinoma | 0.834 (0.779-0.894) |  |  |  |  |
| Surgery | |  | ＜0.001 |  |  | ＜0.001 |
|  | Yes | Reference |  |  | Reference |  |
|  | No | 3.731 (3.580-3.888) |  |  | 3.925(3.749-4.108) |  |
| Radiotherapy | |  | ＜0.001 |  |  | 0.018 |
|  | Yes | Reference |  |  | Reference |  |
|  | None/Unknown | 1228 (1.132-1.332) |  |  | 1.105 (1.017-1.201) |  |
| Chemotherapy | |  | ＜0.001 |  |  | ＜0.001 |
|  | Yes | Reference |  |  | Reference |  |
|  | None/Unknown | 1.454 (1.395-1.515) |  |  | 1.136 (1.085-1.189) |  |
| ^＊^Asian, Pacific Islander, American Indian or Alaska Native | | | | | | |

**Supplementary Table 7. Univariate and multivariate analysis of cancer-specific survival for rectal patients after propensity score matching.**

| **Variables** | | **Univariate analysis** | |  | **Multivariate analysis** | |
| --- | --- | --- | --- | --- | --- | --- |
|  |  | **Odds ratio (95% CI)** | ***p*-value** |  | **Odds ratio (95% CI)** | ***p*-value** |
| Age | |  | ＜0.001 |  |  | ＜0.001 |
|  | 70-74 years | Reference |  |  | Reference |  |
|  | 75-79 years | 0.949 (0.885-1.018) |  |  | 1.056 (0.984-1.133) |  |
|  | 80-84 years | 1.131 (1.055-1.213) |  |  | 1.175 (1.094-1.261) |  |
|  | 85+ years | 1.310 (1.231-1.395) |  |  | 1.369 (1.279-1.466) |  |
| Sex | |  | ＜0.001 |  |  | ＜0.001 |
|  | Male | Reference |  |  | Reference |  |
|  | Female | 0.875 (0.837-0.916) |  |  | 0.911 (0.868-0.957) |  |
| Year of diagnosis | |  | 0.066 |  |  |  |
|  | 2010-2013 | Reference |  |  |  |  |
|  | 2014-2017 | 1.044 (0.997-1.093) |  |  |  |  |
| Race | |  | ＜0.001 |  |  | ＜0.001 |
|  | White | Reference |  |  | Reference |  |
|  | Black | 0.703 (0.659-0.749) |  |  | 1.103 (1.032-1.179) |  |
|  | Others^＊^ | 0.592 (0.549-0.638) |  |  | 0.884 (0.818-0.954) |  |
| Marital status | |  | 0.007 |  |  | ＜0.001 |
|  | Married | Reference |  |  | Reference |  |
|  | Single | 1.065 (1.017-1.115) |  |  | 1.129 (1.075-1.187) |  |
| Grade | |  | ＜0.001 |  |  | ＜0.001 |
|  | Well differentiated (Grade I) | Reference |  |  | Reference |  |
|  | Moderately differentiated (Grade II) | 1.680 (1.482-1.905) |  |  | 1.411 (1.301-1.530) |  |
|  | Poorly differentiated (Grade III) | 1.934 (1.786-2.094) |  |  | 2.219 (2.033-2.422) |  |
|  | Undifferentiated (Grade IV) | 2.276 (2.087-2.483) |  |  | 2.344 (2.063-2.662) |  |
| Histology | |  | ＜0.001 |  |  |  |
|  | Adenocarcinoma | Reference |  |  |  |  |
|  | Mucinous adenocarcinoma | 0.795 (0.732-0.865) |  |  |  |  |
| Surgery | |  | ＜0.001 |  |  | ＜0.001 |
|  | Yes | Reference |  |  | Reference |  |
|  | No | 4.328 (4.117-4.549) |  |  | 4.740(4.487-5.006) |  |
| Radiotherapy | |  | 0.027 |  |  |  |
|  | Yes | Reference |  |  |  |  |
|  | None/Unknown | 1.109 (1.012-1.216) |  |  |  |  |
| Chemotherapy | |  | ＜0.001 |  |  | ＜0.001 |
|  | Yes | Reference |  |  | Reference |  |
|  | None/Unknown | 1.142 (1.089-1.196) |  |  | 0.911 (0.865-0.960) |  |
| ^＊^Asian, Pacific Islander, American Indian or Alaska Native | | | | | | |

**Univariate and multivariate Cox analyses in patients underwent surgery after PSM according to tumor location**

**Supplementary Table 8. Univariate and multivariate analysis of overall survival for colon patients who underwent surgery after propensity score matching.**

| **Variables** | | **Univariate analysis** | |  | **Multivariate analysis** | |
| --- | --- | --- | --- | --- | --- | --- |
|  |  | **Odds ratio (95% CI)** | ***p*-value** |  | **Odds ratio (95% CI)** | ***p*-value** |
| Age | |  | ＜0.001 |  |  | ＜0.001 |
|  | 70-74 years | Reference |  |  | Reference |  |
|  | 75-79 years | 1.218 (1.074-1.382) |  |  | 1.236 (1.088-1.404) |  |
|  | 80-84 years | 1.546 (1.360-1.756) |  |  | 1.573 (1.380-1.793) |  |
|  | 85+ years | 2.186 (1.947-2.455) |  |  | 2.324 (2.042-2.646) |  |
| Sex | |  | 0.002 |  |  | ＜0.001 |
|  | Male | Reference |  |  | Reference |  |
|  | Female | 0.886 (0.820-0.957) |  |  | 0.767 (0.707-0.833) |  |
| Year of diagnosis | |  | 0.582 |  |  |  |
|  | 2010-2013 | Reference |  |  |  |  |
|  | 2014-2017 | 1.024 (0.942-1.112) |  |  |  |  |
| Race | |  | ＜0.001 |  |  | ＜0.001 |
|  | White | Reference |  |  | Reference |  |
|  | Black | 0.954 (0.870-1.045) |  |  | 1.204 (1.090-1.331) |  |
|  | Others^＊^ | 0.781 (0.702-0.870) |  |  | 0.923 (0.821-1.037) |  |
| Marital status | |  | ＜0.001 |  |  | ＜0.001 |
|  | Married | Reference |  |  | Reference |  |
|  | Single | 1.259 (1.162-1.363) |  |  | 1.167 (1.074-1.269) |  |
| AJCC T stage | |  | ＜0.001 |  |  | ＜0.001 |
|  | T1 | Reference |  |  | Reference |  |
|  | T2 | 1.196 (1.007-1.419) |  |  | 1.146 (0.962-1.365) |  |
|  | T3 | 1.390 (1.217-1.588) |  |  | 1.412 (1.137-1.753) |  |
|  | T4 | 2.250 (1.951-2.594) |  |  | 1.883 (1.508-2.351) |  |
| AJCC N stage | |  | ＜0.001 |  |  | ＜0.001 |
|  | N0 | Reference |  |  | Reference |  |
|  | N1 | 1.100 (1.008-1.201) |  |  | 1.136 (1.010-1.278) |  |
|  | N2 | 1.725 (1.564-1.904) |  |  | 1.655 (1.453-1.885) |  |
| AJCC M stage | |  | ＜0.001 |  |  | ＜0.001 |
|  | M0 | Reference |  |  | Reference |  |
|  | M1 | 2.011 (1.830-2.210) |  |  | 1.868 (1.659-2.104) |  |
| AJCC stage | |  | ＜0.001 |  |  |  |
|  | I | Reference |  |  |  |  |
|  | II | 1.208 (1.065-1.370) |  |  |  |  |
|  | III | 1.518 (1.357-1.697) |  |  |  |  |
|  | IV | 1.900 (1.561-2.313) |  |  |  |  |
| Perineural invasion | |  | ＜0.001 |  |  | ＜0.001 |
|  | No | Reference |  |  | Reference |  |
|  | Yes | 1.790 (1.621-1.978) |  |  | 1.585 (1.427-1.759) |  |
| Tumor deposits | |  | ＜0.001 |  |  | ＜0.001 |
|  | No | Reference |  |  | Reference |  |
|  | Yes | 1.358 (1.270-1.452) |  |  | 1.361 (1.272-1.455) |  |
| Grade | |  | ＜0.001 |  |  | ＜0.001 |
|  | Well differentiated (Grade I) | Reference |  |  | Reference |  |
|  | Moderately differentiated (Grade II) | 1.205 (1.074-1.353) |  |  | 1.124 (0.996-1.267) |  |
|  | Poorly differentiated (Grade III) | 1.732 (1.530-1.961) |  |  | 1.361 (1.197-1.548) |  |
|  | Undifferentiated (Grade IV) | 1.944 (1.659-2.277) |  |  | 1.618 (1.375-1.904) |  |
| Histology | |  | 0.013 |  |  |  |
|  | Adenocarcinoma | Reference |  |  |  |  |
|  | Mucinous adenocarcinoma | 1.151 (1.030-1.285) |  |  |  |  |
| Chemotherapy | |  | ＜0.001 |  |  | ＜0.001 |
|  | Yes | Reference |  |  | Reference |  |
|  | None/Unknown | 1.221 (1.130-1.319) |  |  | 1.187 (1.082-1.301) |  |
| ^＊^Asian, Pacific Islander, American Indian or Alaska Native | | | | | | |

**Supplementary Table 9. Univariate and multivariate analysis of cancer-specific survival for colon patients underwent surgery after propensity score matching.**

| **Variables** | | **Univariate analysis** | |  | **Multivariate analysis** | |
| --- | --- | --- | --- | --- | --- | --- |
|  |  | **Odds ratio (95% CI)** | ***p*-value** |  | **Odds ratio (95% CI)** | ***p*-value** |
| Age | |  | ＜0.001 |  |  | ＜0.001 |
|  | 70-74 years | Reference |  |  | Reference |  |
|  | 75-79 years | 1.183 (0.952-1.292) |  |  | 1.095 (0.938-1.278) |  |
|  | 80-84 years | 1.232 (1.052-1.443) |  |  | 1.346 (1.145-1.583) |  |
|  | 85+ years | 1.602 (1.390-1.847) |  |  | 1.917 (1.632-2.251) |  |
| Sex | |  | 0.006 |  |  | ＜0.001 |
|  | Male | Reference |  |  | Reference |  |
|  | Female | 0.870 (0.787-0.960) |  |  | 0.818 (0.737-0.908) |  |
| Year of diagnosis | |  | 0.962 |  |  |  |
|  | 2010-2013 | Reference |  |  |  |  |
|  | 2014-2017 | 1.003 (0.905-1.111) |  |  |  |  |
| Race | |  | 0.001 |  |  | 0.001 |
|  | White | Reference |  |  | Reference |  |
|  | Black | 0.964 (0.858-1.083) |  |  | 1.179 (1.040-1.336) |  |
|  | Others^＊^ | 0.697 (0.604-0.805) |  |  | 0.872 (0.747-1.017) |  |
| Marital status | |  | ＜0.001 |  |  | 0.004 |
|  | Married | Reference |  |  | Reference |  |
|  | Single | 1.232 (1.113-1.365) |  |  | 1.170 (1.052-1.302) |  |
| AJCC T stage | |  | ＜0.001 |  |  | ＜0.001 |
|  | T1 | Reference |  |  | Reference |  |
|  | T2 | 1.235 (0.960-1.589) |  |  | 1.103 (0.856-1.423) |  |
|  | T3 | 1.819 (1.498-2.208) |  |  | 1.315 (1.073-1.610) |  |
|  | T4 | 3.352 (2.741-4.100) |  |  | 1.820 (1.468-2.258) |  |
| AJCC N stage | |  | ＜0.001 |  |  | ＜0.001 |
|  | N0 | Reference |  |  | Reference |  |
|  | N1 | 1.394 (1.241-1.565) |  |  | 1.149 (1.015-1.301) |  |
|  | N2 | 2.557 (2.264-2.888) |  |  | 1.569 (1.369-1.798) |  |
| AJCC M stage | |  | ＜0.001 |  |  | ＜0.001 |
|  | M0 | Reference |  |  | Reference |  |
|  | M1 | 2.840 (2.547-3.169) |  |  | 2.235 (1.989-2.511) |  |
| AJCC stage | |  | ＜0.001 |  |  |  |
|  | I | Reference |  |  |  |  |
|  | II | 1.385 (1.148-1.670) |  |  |  |  |
|  | III | 2.391 (2.029-2.817) |  |  |  |  |
|  | IV | 3.042 (2.367-3.909) |  |  |  |  |
| Perineural invasion | |  | ＜0.001 |  |  | ＜0.001 |
|  | No | Reference |  |  | Reference |  |
|  | Yes | 2.386 (2.125-2.680) |  |  | 1.822 (1.613-2.058) |  |
| Tumor deposits | |  | ＜0.001 |  |  | ＜0.001 |
|  | No | Reference |  |  | Reference |  |
|  | Yes | 1.409 (1.327-1.496) |  |  | 1.407 (1.325-1.494) |  |
| Grade | |  | ＜0.001 |  |  | ＜0.001 |
|  | Well differentiated (Grade I) | Reference |  |  | Reference |  |
|  | Moderately differentiated (Grade II) | 1.663 (1.405-1.968) |  |  | 1.359 (1.143-1.616) |  |
|  | Poorly differentiated (Grade III) | 2.413 (2.022-2.881) |  |  | 1.667 (1.390-2.000) |  |
|  | Undifferentiated (Grade IV) | 2.940 (2.380-3.631) |  |  | 2.109 (1.699-2.619) |  |
| Histology | |  | 0.021 |  |  |  |
|  | Adenocarcinoma | Reference |  |  |  |  |
|  | Mucinous adenocarcinoma | 1.179 (1.025-1.356) |  |  |  |  |
| Chemotherapy | |  | ＜0.001 |  |  |  |
|  | Yes | Reference |  |  |  |  |
|  | None/Unknown | 0.821 (0.745-0.906) |  |  |  |  |
| ^＊^Asian, Pacific Islander, American Indian or Alaska Native | | | | | | |

**Supplementary Table 10. Univariate and multivariate analysis of overall survival for rectal patients who underwent surgery after propensity score matching.**

| **Variables** | | **Univariate analysis** | |  | **Multivariate analysis** | |
| --- | --- | --- | --- | --- | --- | --- |
|  |  | **Odds ratio (95% CI)** | ***p*-value** |  | **Odds ratio (95% CI)** | ***p*-value** |
| Age | |  | ＜0.001 |  |  | ＜0.001 |
|  | 70-74 years | Reference |  |  | Reference |  |
|  | 75-79 years | 1.199 (1.032-1.392) |  |  | 1.220 (1.047-1.421) |  |
|  | 80-84 years | 1.442 (1.247-1.667) |  |  | 1.460 (1.255-1.698) |  |
|  | 85+ years | 2.414 (2.097-2.779) |  |  | 2.306 (1.975-2.691) |  |
| Sex | |  | ＜0.001 |  |  | ＜0.001 |
|  | Male | Reference |  |  | Reference |  |
|  | Female | 0.786 (0.714-0.866) |  |  | 0.705 (0.634-0.783) |  |
| Year of diagnosis | |  | 0.336 |  |  |  |
|  | 2010-2013 | Reference |  |  |  |  |
|  | 2014-2017 | 1. 054 (0.947-1.174) |  |  |  |  |
| Race | |  | ＜0.001 |  |  | ＜0.001 |
|  | White | Reference |  |  | Reference |  |
|  | Black | 1.145 (0.970-1.351) |  |  | 1.281 (1.080-1.520) |  |
|  | Others^＊^ | 0.708 (0.609-0.824) |  |  | 0.853 (0.731-0.996) |  |
| Marital status | |  | ＜0.001 |  |  | ＜0.001 |
|  | Married | Reference |  |  | Reference |  |
|  | Single | 1.199 (1.089-1.319) |  |  | 1.220 (1.098-1.356) |  |
| AJCC T stage | |  | ＜0.001 |  |  | ＜0.001 |
|  | T1 | Reference |  |  | Reference |  |
|  | T2 | 1.000 (0.841-1.189) |  |  | 0.947 (0.817-1.162) |  |
|  | T3 | 1.307 (1.134-1.506) |  |  | 1.389 (1.085-1.778) |  |
|  | T4 | 1.738 (1.450-2.083) |  |  | 1.556 (1.186-2.040) |  |
| AJCC N stage | |  | ＜0.001 |  |  | ＜0.001 |
|  | N0 | Reference |  |  | Reference |  |
|  | N1 | 1.168 (1.047-1.303) |  |  | 1.181 (1.051-1.327) |  |
|  | N2 | 1.530 (1.325-1.767) |  |  | 1.282 (1.094-1.502) |  |
| AJCC M stage | |  | ＜0.001 |  |  | ＜0.001 |
|  | M0 | Reference |  |  | Reference |  |
|  | M1 | 1.651 (1.417-1.924) |  |  | 1.521 (1.288-1.796) |  |
| AJCC stage | |  | ＜0.001 |  |  |  |
|  | I | Reference |  |  |  |  |
|  | II | 1.192 (1.047-1.358) |  |  |  |  |
|  | III | 1.411 (1.251-1.592) |  |  |  |  |
|  | IV | 1.714 (1.267-2.318) |  |  |  |  |
| Perineural invasion | |  | ＜0.001 |  |  | ＜0.001 |
|  | No | Reference |  |  | Reference |  |
|  | Yes | 1.951 (1.706-2.230) |  |  | 1.661 (1.446-1.907) |  |
| Tumor deposits | |  | ＜0.001 |  |  | ＜0.001 |
|  | No | Reference |  |  | Reference |  |
|  | Yes | 1.364 (1.258-1.479) |  |  | 1.357 (1.252-1.471) |  |
| Grade | |  | ＜0.001 |  |  | ＜0.001 |
|  | Well differentiated (Grade I) | Reference |  |  | Reference |  |
|  | Moderately differentiated (Grade II) | 1.098 (0.940-1.281) |  |  | 1.008 (0.861-1.182) |  |
|  | Poorly differentiated (Grade III) | 1.358 (1.124-1.640) |  |  | 1.173 (0.963-1.428) |  |
|  | Undifferentiated (Grade IV) | 1.873 (1.374-2.544) |  |  | 1.834 (1.339-2.513) |  |
| Histology | |  | 0.007 |  |  | 0.025 |
|  | Adenocarcinoma | Reference |  |  | Reference |  |
|  | Mucinous adenocarcinoma | 1.274 (1.067-1.520) |  |  | 1.229 (1.027-1.470) |  |
| Radiotherapy | |  | ＜0.001 |  |  | 0.024 |
|  | Yes | Reference |  |  | Reference |  |
|  | None/Unknown | 1.493 (1.354-1.647) |  |  | 1.178 (1.021-1.358) |  |
| Chemotherapy | |  | ＜0.001 |  |  | ＜0.001 |
|  | Yes | Reference |  |  | Reference |  |
|  | None/Unknown | 1.464 (1.329-1.611) |  |  | 1.306 (1.1.27-1.512) |  |
| ^＊^Asian, Pacific Islander, American Indian or Alaska Native | | | | | | |

**Supplementary Table 11. Univariate and multivariate analysis of cancer-specific survival for rectal patients underwent surgery after propensity score matching.**

| **Variables** | | **Univariate analysis** | |  | **Multivariate analysis** | |
| --- | --- | --- | --- | --- | --- | --- |
|  |  | **Odds ratio (95% CI)** | ***p*-value** |  | **Odds ratio (95% CI)** | ***p*-value** |
| Age | |  | ＜0.001 |  |  | ＜0.001 |
|  | 70-74 years | Reference |  |  | Reference |  |
|  | 75-79 years | 1.150 (0.952-1.389) |  |  | 1.211 (0.999-1.468) |  |
|  | 80-84 years | 1.218 (1.010-1.469) |  |  | 1.344 (1.106-1.634) |  |
|  | 85+ years | 1.760 (1.463-2.118) |  |  | 1.938 (1.582-2.347) |  |
| Sex | |  | ＜0.001 |  |  | ＜0.001 |
|  | Male | Reference |  |  | Reference |  |
|  | Female | 0.779 (0.686-0.886) |  |  | 0.696 (0.605-0.800) |  |
| Year of diagnosis | |  | 0.905 |  |  |  |
|  | 2010-2013 | Reference |  |  |  |  |
|  | 2014-2017 | 1.009 (0.877-1.160) |  |  |  |  |
| Race | |  | 0.014 |  |  |  |
|  | White | Reference |  |  |  |  |
|  | Black | 1.131 (0.906-1.412) |  |  |  |  |
|  | Others^＊^ | 0.775 (0.637-0.942) |  |  |  |  |
| Marital status | |  | 0.001 |  |  | ＜0.001 |
|  | Married | Reference |  |  | Reference |  |
|  | Single | 1.246 (1.097-1.416) |  |  | 1.333 (1.159-1.533) |  |
| AJCC T stage | |  | ＜0.001 |  |  | ＜0.001 |
|  | T1 | Reference |  |  | Reference |  |
|  | T2 | 1.111 (0.856-1.441) |  |  | 1.017 (0.782-1.323) |  |
|  | T3 | 1.860 (1.505-2.298) |  |  | 1.496 (1.193-1.874) |  |
|  | T4 | 2.700 (2.101-3.471) |  |  | 1.816 (1.391-2.371) |  |
| AJCC N stage | |  | ＜0.001 |  |  | ＜0.001 |
|  | N0 | Reference |  |  | Reference |  |
|  | N1 | 1.625 (1.409-1.873) |  |  | 1.439 (1.238-1.674) |  |
|  | N2 | 2.265 (1.897-2.705) |  |  | 1.574 (1.292-1.917) |  |
| AJCC M stage | |  | ＜0.001 |  |  | ＜0.001 |
|  | M0 | Reference |  |  | Reference |  |
|  | M1 | 2.266 (1.895-2.710) |  |  | 1.766 (1.451-2.148) |  |
| AJCC stage | |  | ＜0.001 |  |  |  |
|  | I | Reference |  |  |  |  |
|  | II | 1.619 (1.334-1.966) |  |  |  |  |
|  | III | 2.419 (2.208-2.886) |  |  |  |  |
|  | IV | 2.500 (1.674-3.733) |  |  |  |  |
| Perineural invasion | |  | ＜0.001 |  |  | ＜0.001 |
|  | No | Reference |  |  | Reference |  |
|  | Yes | 1.951 (1.706-2.230) |  |  | 1.897 (1.603-2.244) |  |
| Tumor deposits | |  | ＜0.001 |  |  | ＜0.001 |
|  | No | Reference |  |  | Reference |  |
|  | Yes | 1.515 (1.381-1.663) |  |  | 1.516 (1.382-1.664) |  |
| Grade | |  | ＜0.001 |  |  | ＜0.001 |
|  | Well differentiated (Grade I) | Reference |  |  | Reference |  |
|  | Moderately differentiated (Grade II) | 1.518 (1.199-1.922) |  |  | 1.264 (0.994-1.607) |  |
|  | Poorly differentiated (Grade III) | 2.138 (1.631-2.801) |  |  | 1.486 (1.123-1.965) |  |
|  | Undifferentiated (Grade IV) | 2.861 (1.903-4.302) |  |  | 2.398 (1.585-3.629) |  |
| Histology | |  | 0.001 |  |  | 0.012 |
|  | Adenocarcinoma | Reference |  |  | Reference |  |
|  | Mucinous adenocarcinoma | 1.459 (1.170-1.819) |  |  | 1.332 (1.064-1.668) |  |
| Radiotherapy | |  | 0.002 |  |  | 0.018 |
|  | Yes | Reference |  |  | Reference |  |
|  | None/Unknown | 1.230 (1.082-1.398) |  |  | 1.241 (1.038-1.483) |  |
| Chemotherapy | |  | 0.627 |  |  |  |
|  | Yes | Reference |  |  |  |  |
|  | None/Unknown | 1.032 (0.909-1.173) |  |  |  |  |
| ^＊^Asian, Pacific Islander, American Indian or Alaska Native | | | | | | |

**Survival comparisons of different treatment patterns of rectal patients underwent surgery after PSM in different age groups**

**Supplementary Table 12. Pairwise** **comparisons for different treatment patterns of rectal patients underwent surgery**

| Outcome* |  | Surgery+RT | Surgery+CT | Surgery+CRT |
| --- | --- | --- | --- | --- |
| OS of patients with ages 70–74 |  |  |  |  |
|  | Surgery alone | **1.725 (1.029, 2.839)** | 1.357 (0.958, 1.921) | **2.623 (1.920, 3.583)** |
|  | Surgery+RT | - | 0.771 (0.472, 1.260) | 1.460 (0.918, 2.322) |
|  | Surgery+CT | - | - | **1.932 (1.481, 2.520)** |
| OS of patients with ages 75–79 |  |  |  |  |
|  | Surgery alone | 1.254 (0.768, 2.049) | **1.854 (1.331, 2.584)** | **2.367 (1.852, 3.026)** |
|  | Surgery+RT | - | 1.488 (0.881, 2.514) | **1.917 (1.193, 3.080)** |
|  | Surgery+CT | - | - | 1.242 (0.916, 1.683) |
| OS of patients with ages 80–84 |  |  |  |  |
|  | Surgery alone | 1.523 (0.907, 2.559) | 1.213 (0.871, 1.687) | **2.026 (1.628, 2.521)** |
|  | Surgery+RT | - | 0.782 (0.443, 1.381) | 1.245 (0.747, 2.074) |
|  | Surgery+CT | - | - | **1.678 (1.218, 2.312)** |
| OS of patients with ages 85+ |  |  |  |  |
|  | Surgery alone | 0.731 (0.503, 1.064) | 1.605 (0.994, 2.593) | **1.840 (1.456, 2.326)** |
|  | Surgery+RT | - | **2.210 (1.231, 3.967)** | **2.569 (1.708, 3.863)** |
|  | Surgery+CT | - | - | 1.133 (0.685, 1.874) |
| CSS of patients with ages 70–74 |  |  |  |  |
|  | Surgery alone | 1.716 (0.880, 3.347) | 0.968 (0.634, 1.480) | **2.469 (1.652, 3.691)** |
|  | Surgery+RT | - | 0.557 (0.302, 1.030) | 1.460 (0.918,2.322) |
|  | Surgery+CT | - | - | 1.346 (0.950,1.907) |
| CSS of patients with ages 75–79 |  |  |  |  |
|  | Surgery alone | 1.206 (0.665, 2.189) | **1.546 (1.048, 2.280)** | **2.122 (1.573, 2863)** |
|  | Surgery+RT | - | 1.270 (0.681, 2.368) | 1.769 (0.999, 3.134) |
|  | Surgery+CT | - | - | **2.541 (1.871, 3.450)** |
| CSS of patients with ages 80–84 |  |  |  |  |
|  | Surgery alone | 1.760 (0.851, 3.639) | 1.045 (0.699, 1.562) | **1.875 (1.418, 2.479)** |
|  | Surgery+RT | - | 0.586 (0.270, 1.271) | 1.016 (0.496, 2.078) |
|  | Surgery+CT | - | - | **1.792 (1.217, 2.637)** |
| CSS of patients with ages 85+ |  |  |  |  |
|  | Surgery alone | **0.534 (0.351, 0.812)** | 1.012 (0.594, 1.726) | **1.513 (1.122, 2.040)** |
|  | Surgery+RT | - | **1.917 (1.011, 3.638)** | **2.906 (1.832, 4.610)** |
|  | Surgery+CT | - | - | 1.463 (0.832, 2.572) |
| *Odds ratio of horizontal treatment over vertical treatment  OS, overall survival; CSS, cancer-specific survival; CRT, chemoradiotherapy; CT, chemotherapy; RT, radiotherapy. | | | | |
