# Supplementary material for: Real-world analysis of survival benefit of surgery and adjuvant therapy in elderly patients with colorectal cancer
Source: Sci Rep. 2023 Sep 8;13:14866. doi: 10.1038/s41598-023-41713-1 (PMC10491681; doi:10.1038/s41598-023-41713-1)
Supplement: Supplementary file 2 — Supplementary Figures. [file 41598_2023_41713_MOESM2_ESM.docx]

**Real-world analysis of survival benefit of surgery and adjuvant therapy in elderly patients with colorectal cancer**

Zhang et al.

(Supplementary figures)

**Results**

**
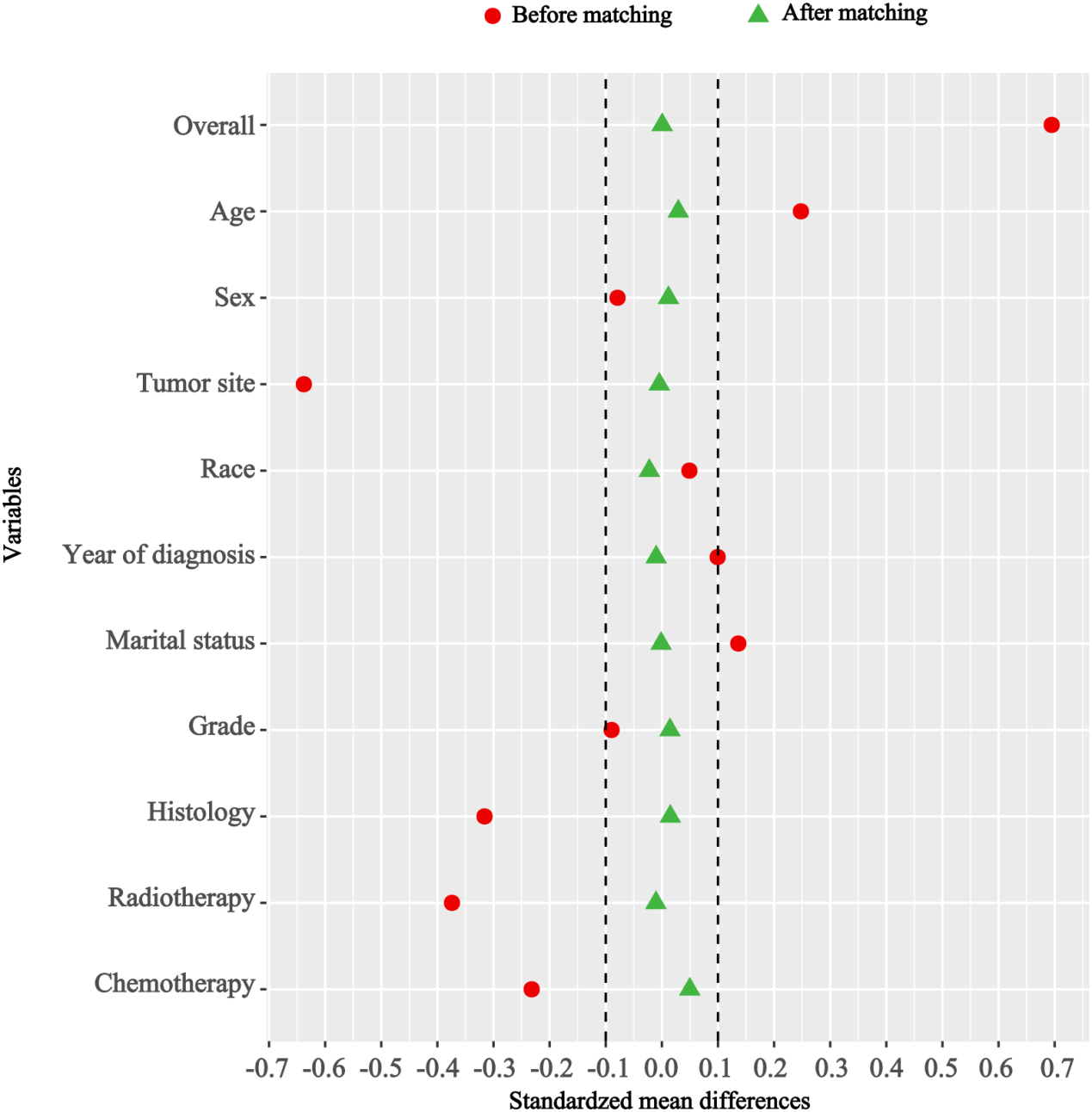
**

**Supplementary Figure 1. Standardized mean differences before and after propensity score matching.** Mean standardized difference ≥ 0.1 is considered significant

**Survival outcomes in surgical and nonsurgical patients after PSM according to tumor location**

In colon cancer patients, the 5-year OS rates for patients underwent surgery with ages 70–74, 75–79, 80–84, and ≥85 were 61.3%, 59.8%, 51.1%, and 39.2%, which were clearly better than patients treated with no-surgery (**Supplementary Figure 2, A-D**). Similarly, in patients treated with surgery, the 5-year CSS rates for patients with ages 70–74, 75–79, 80–84, and ≥85 were 71.6%, 68.9%, 63.4%, and 60.1%, which were significantly better prognosis than patients treated with no-surgery (**Supplementary Figure 2, E-H**).

In rectal cancer patients, the 5-year OS rates for patients underwent surgery with ages 70–74, 75–79, 80–84, and ≥85 were 62.1%, 60.1%, 53.2%, and 39.8%, which were clearly better than patients treated with no-surgery (**Supplementary Figure 3, A-D**). Similarly, in patients treated with surgery, the 5-year CSS rates for patients with ages 70–74, 75–79, 80–84, and ≥85 were 76.4%, 73.6%, 67.4%, and 60.2%, which were significantly better prognosis than patients treated with no-surgery (**Supplementary Figure 3, E-H**). **
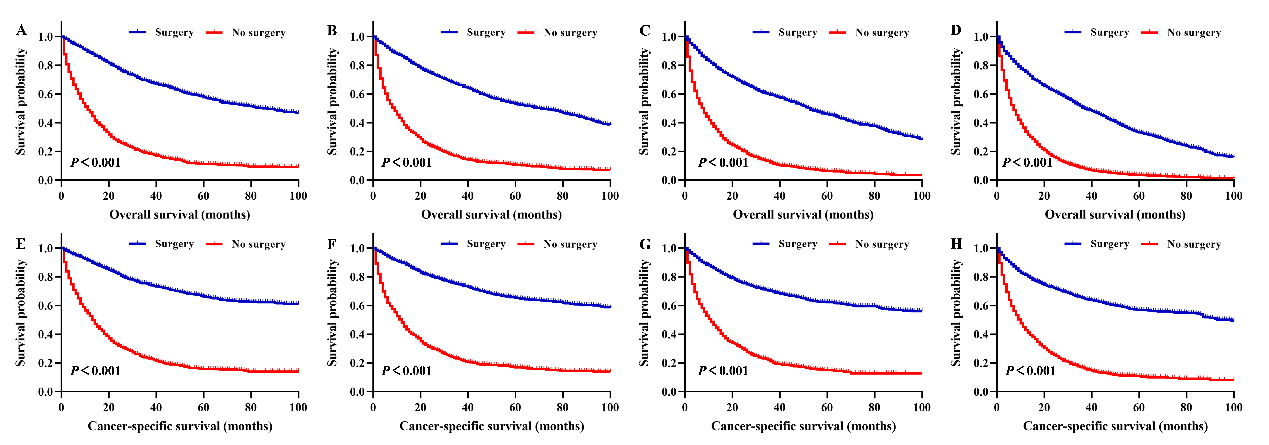
**

**Supplementary Figure 2. According to whether or not surgery, Kaplan-Meier survival analysis of colon cancer patients after propensity score matching.** Overall survival for patients 70-74 years old (A), 75–79 years old (B), 80–84 years old (C), and 85+ years old (D). Cancer-specific survival for patients 70-74 years old (E), 75–79 years old (F), 80–84 years old (G), and 85+ years old (H).

**
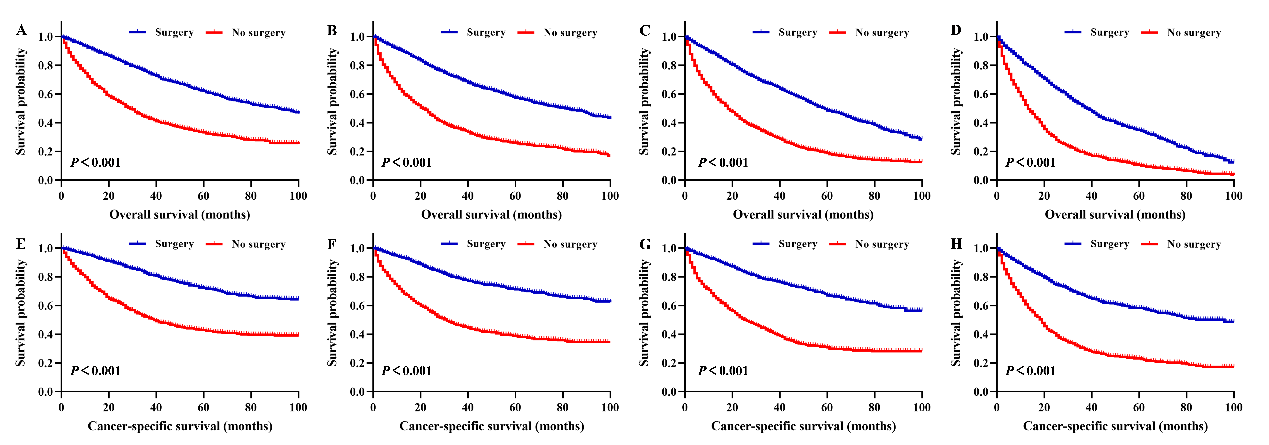
**

**Supplementary Figure 3. According to whether or not surgery, Kaplan-Meier survival analysis of rectal cancer patients after propensity score matching.** Overall survival for patients 70-74 years old (A), 75–79 years old (B), 80–84 years old (C), and 85+ years old (D). Cancer-specific survival for patients 70-74 years old (E), 75–79 years old (F), 80–84 years old (G), and 85+ years old (H).

**Survival outcomes of patients underwent surgery with different tumor stages and nonsurgical patients after PSM according to tumor location**

In colon cancer patients, the 5-year OS rates for patients underwent surgery with I stage, II stage, III stage, and IV stage were 61.9%, 58.6%, 53.4%, and 9.0%, which were clearly better than patients treated with no-surgery (**Supplementary Figure 4, A-D**). Similarly, in patients treated with surgery, the 5-year CSS rates for patients with I stage, II stage, III stage, and IV stage were 89.1%, 79.9%, 62.2%, and 17.1%, which were significantly better prognosis than patients treated with no-surgery (**Supplementary Figure 4, E-H**).

In rectal cancer patients, the 5-year OS rates for patients underwent surgery with I stage, II stage, and III stage were 61.1%, 57.9%, and 49.3%, which were clearly better than patients treated with no-surgery (**Supplementary Figure 5, A-C**). Similarly, in patients treated with surgery, the 5-year CSS rates for patients with I stage, II stage, and III stage were 82.4%, 67.5%, and 60.9%, which were significantly better prognosis than patients treated with no-surgery (**Supplementary Figure 5, E-G**). And, the 5-year OS were 20.1% and 27.2% for patients underwent surgery with IV stage and no-surgery, respectively (P = 0.892, **Supplementary Figure 5D**). The 5-year CSS were 24.2% and 39.1% for patients underwent surgery with IV stage and no-surgery, respectively (P = 0.019, **Supplementary Figure 5H**).

**
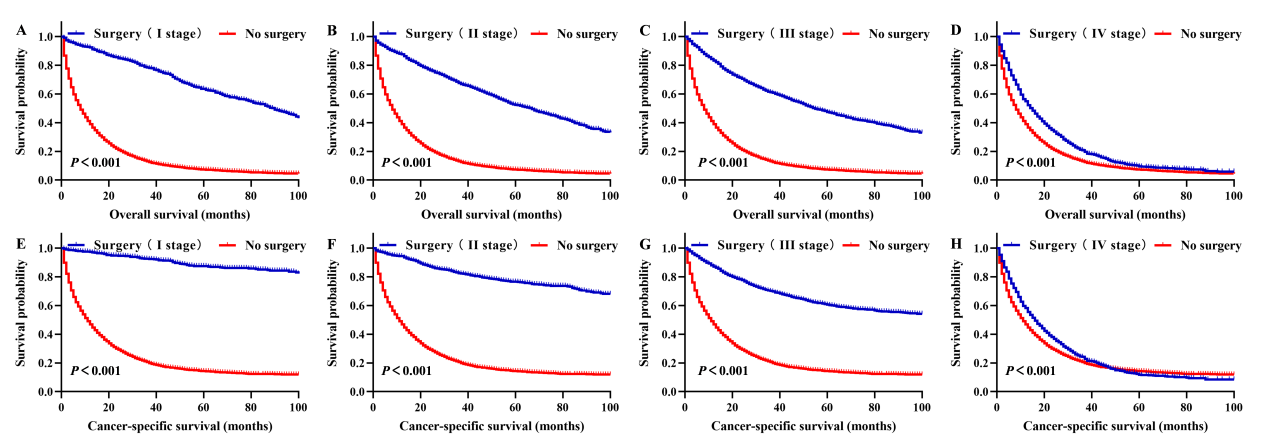
**

**Supplementary Figure 4. According to patients underwent surgery with different tumor stages, Kaplan-Meier survival analysis of colon cancer patients after propensity score matching (surgery vs. no-surgery).** Overall survival for patients underwent surgery with I stage (A), II stage (B), III stage (C), and IV stage (D). Cancer-specific survival for patients underwent surgery with I stage (E), II stage (F), III stage (G), and IV stage (H).

**
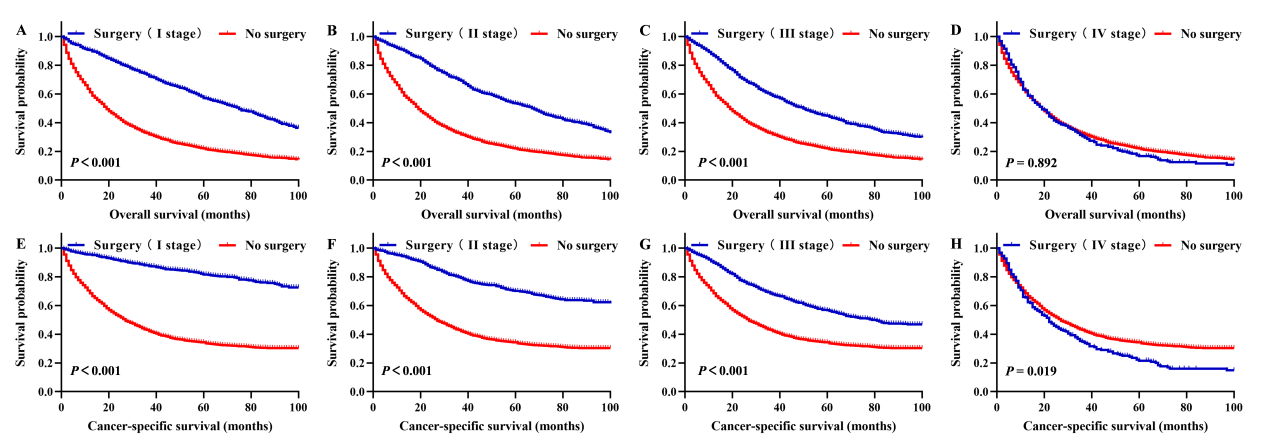
**

**Supplementary Figure 5. According to patients underwent surgery with different tumor stages, Kaplan-Meier survival analysis of rectal cancer patients after propensity score matching (surgery vs. no-surgery).** Overall survival for patients underwent surgery with I stage (A), II stage (B), III stage (C), and IV stage (D). Cancer-specific survival for patients underwent surgery with I stage (E), II stage (F), III stage (G), and IV stage (H).
